# Mechanistic Modeling of Lys745 Sulfonylation in EGFR C797S Reveals Chemical Determinants for Inhibitor Activity and Discriminates Reversible from Irreversible Agents

**DOI:** 10.1021/acs.jcim.2c01586

**Published:** 2023-02-10

**Authors:** Kemel Arafet, Laura Scalvini, Francesca Galvani, Sergio Martí, Vicent Moliner, Marco Mor, Alessio Lodola

**Affiliations:** † Dipartimento di Scienze degli Alimenti e del Farmaco, Università degli Studi di Parma, Parco Area delle Scienze 27/A, I- 43124 Parma, Italy; ‡ BioComp Group, Institute of Advanced Materials (INAM), Universitat Jaume I, 12071 Castelló, Spain; § Microbiome Research Hub, University of Parma, Parco Area delle Scienze 11/A, I-43124 Parma, Italy

## Abstract

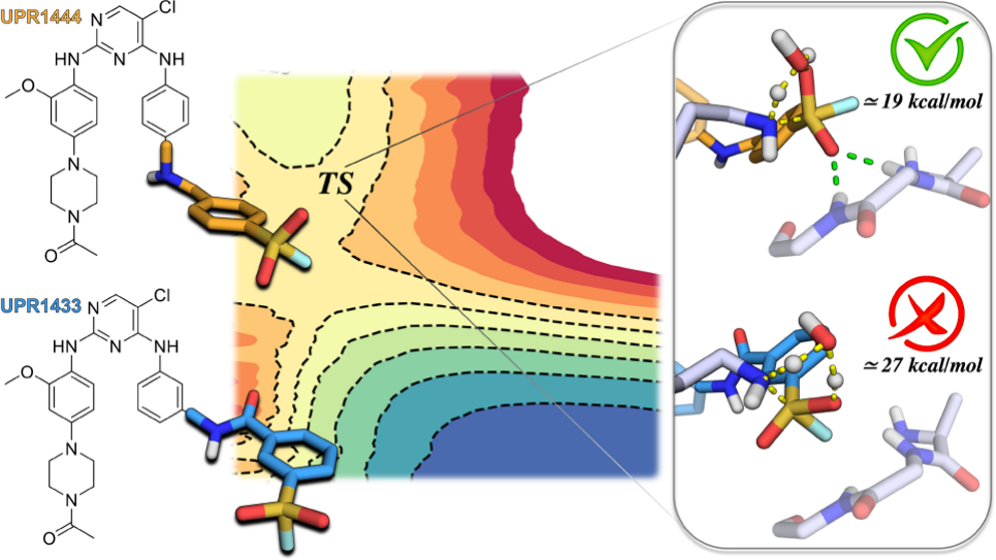

Targeted covalent inhibitors hold promise for drug discovery, particularly for kinases. Targeting the catalytic lysine of epidermal growth factor receptor (EGFR) has attracted attention as a new strategy to overcome resistance due to the emergence of C797S mutation. Sulfonyl fluoride derivatives able to inhibit EGFR^L858R/T790M/C797S^ by sulfonylation of Lys745 have been reported. However, atomistic details of this process are still poorly understood. Here, we describe the mechanism of inhibition of an innovative class of compounds that covalently engage the catalytic lysine of EGFR, through a sulfur(VI) fluoride exchange (SuFEx) process, with the help of hybrid quantum mechanics/molecular mechanics (QM/MM) and path collective variables (PCVs) approaches. Our simulations identify the chemical determinants accounting for the irreversible activity of agents targeting Lys745 and provide hints for the further optimization of sulfonyl fluoride agents.

## Introduction

Epidermal growth factor receptor (EGFR) is a transmembrane protein, featuring an extracellular EGF binding domain and an intracellular tyrosine kinase domain, responsible for the transduction of signals promoting cell proliferation.^1^ Hyper-activating mutations in the kinase domain of EGFR, at the level of exon 19 (del19) or exon 21 (L858R mutation), have been observed in at least 50% of non-small-cell lung cancer (NSCLC).^2^ These alterations promote NSCLC insurgence and progression as their presence results in the constitutive activation of EGFR.^3^

**Figure 1:**
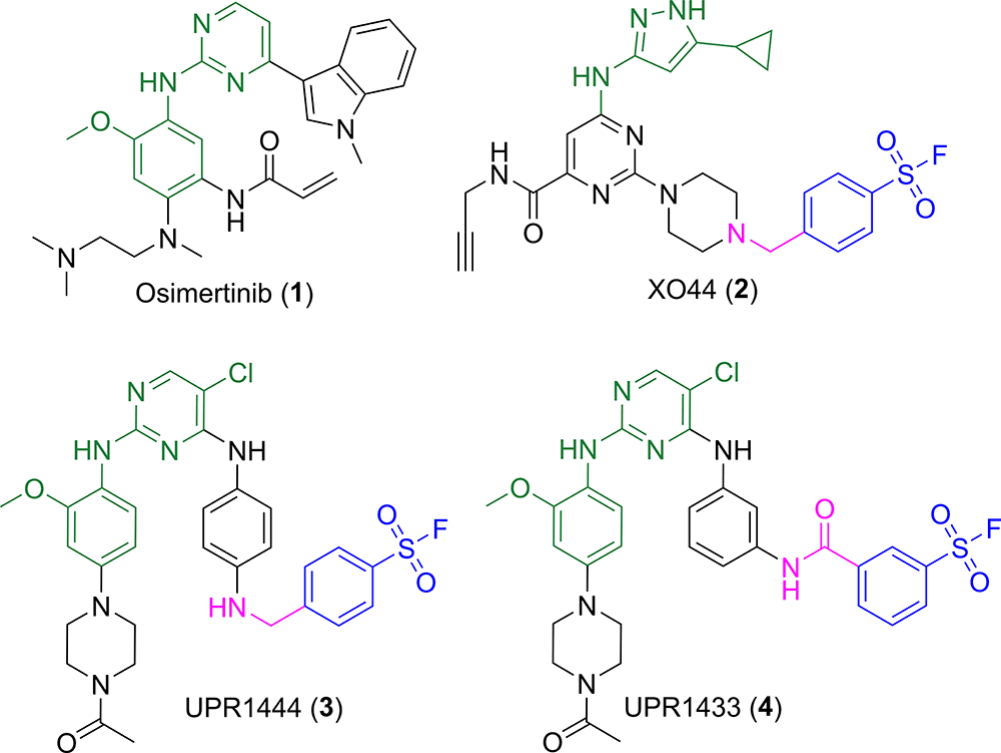
Relevant EGFR inhibitors cited in this work. The reactive warhead of the inhibitors is represented in blue, the linker connecting the warhead and the hinge region binding scaffold is colored in pink, and the hinge region binding scaffold is highlighted in green.

Osimertinib (**1**, Figure 1) is a targeted covalent inhibitor (TCI) bearing an acrylamide warhead^4^ used in first line for patients affected by EGFR mutation-positive NSCLC and in second line for patients harboring the acquired mutation T790M, emerged after treatment with first-generation inhibitors.^5^ Despite its initial efficacy, the use of osimertinib is hampered by the acquired resistance driven by C797S mutation.^6^ The lack of a thiol sidechain at position 797 prevents the formation of a covalent bond between the acrylamide group of osimertinib and EGFR,^7,8^ allowing EGFR itself to escape irreversible inhibition.^9^

Medicinal chemistry efforts have recently demonstrated that the detrimental effect of C797S mutation can be overcome.^10,11^ Compounds able to occupy accessory pockets in the kinase active site of EGFR, forming an extensive network of polar interactions with the catalytic lysine of EGFR (Lys745) and neighboring residues, have been reported to display an exceptional affinity for EGFR C797S variants.^12^ Different compounds exploiting this approach have been described lately,^13^ including the clinical candidate BLU-945, reported to block EGFR^L858R/T790M/C797S^ in animal models.^14^

An alternative strategy to target EGFR variants insensitive to osimertinib is represented by the development of covalent agents targeting a residue distinct from Cys797.^15^ The catalytic lysine of EGFR has attracted growing attention in the discovery of next-generation TCIs in light of its unique chemical and biological properties.^16,17^ Indeed, this lysine is (i) part of the kinase catalytic machinery, and its modification would prevent ATP accommodation leading to enzyme inhibition; (ii) it is surrounded by an environment that can increase its reactivity versus electrophilic warheads; and (iii) it is vital for the phosphorylation activity resulting less prone to mutation than other residues. On the other hand, the conservation of this residue across the whole kinome anticipates that selectivity might be a severe issue.^18^

Sulfonyl fluorides and related S^VI^–F groups are emerging as effective warheads for the covalent targeting of nucleophilic residues other than cysteines and serines for chemical biology applications.^19,20^ Sulfonyl fluorides are well suited to target the lysine residues in proteins as the resulting reaction product (a sulfonamide) is stable to solvolysis, and it is thus expected to ensure irreversible inhibition of the target of interest.^21^ Moreover, S^VI^–F groups react with nucleophiles only in well-organized environments, such as protein binding sites, in which stable dipoles and residues with hydrogen bonding properties can enhance their electrophilicity and favor the expulsion of the fluorine leaving group (LG).^22^ The lack of a well-organized environment prevents sulfur fluoride exchange (SuFEx) process (i.e., sulfonylation) to occur.^23^ This feature is expected to dramatically reduce the cross-reactivity of S^VI^–F groups versus bionucleophiles promoting their use in drug discovery research.

In the context of EGFR inhibition, sulfonyl fluorides have attracted attention thanks to XO44 (**2**, Figure 1).^24^ This compound has been recently reported to sulfonylate Lys745 of EGFR^L858R/T790M/V948R^^25^ and to inhibit EGFR^L858R/T790M/C797S^ more potently than osimertinib.^26^ The lack of selectivity displayed by XO44, inhibiting more than 100 kinases,^25^ hampers covalent targeting of Lys745 as a strategy to search for fourth-generation EGFR inhibitors. On the other hand, the use of an EGFR-selective 2-anilinopirimidine hinge binding scaffold has led to the discovery of UPR1444 (**3**, Figure 1), a second-generation sulfonyl fluoride inhibitor able to block EGFR^L858R/T790M/C797S^ activity through the irreversible sulfonylation of Lys745 that spares the activity of other kinases.^26^ Thus, UPR1444 has emerged as the first covalent inhibitor of EGFR^L858R/T790M/C797S^ endowed with good selectivity, able to inhibit proliferation of cells resistant to osimertinib.

Despite its promising profile, UPR1444 remains a tool compound featuring suboptimal potency in cells. A deep understanding of its mechanism of action, including structure–activity relationships (SARs), is fundamental for a future optimization of the class and for an unbiased evaluation of the catalytic lysine targeting approach. Available data suggest that the chemical requirements to achieve an efficient and irreversible inhibition of EGFR^L858R/T790M/C797S^ through lysine sulfonylation are rather strict. The simple modification of the linker connecting the warhead to the 2-aminopirimidine scaffold (as for UPR1433, **4**, Figure 1) dramatically reduces the rate of EGFR inactivation and the stability of the reaction product. Differently to what was observed for UPR1444, EGFR^L858R/T790M/C797S^ inhibition by UPR1433 is less dependent on the time of preincubation and can be fully reversed by dilution.^26^

In the present work, we applied a hybrid quantum mechanics/molecular mechanics (QM/MM) approach,^27,28^ coupled with the path collective variables (PCVs) method,^29^ to investigate at the atomic level the mechanism of Lys745 sulfonylation in EGFR^L858R/T790M/C797S^. Computational strategies of this kind have allowed to clarify mechanisms of action of enzymes^30,31^ and relevant covalent inhibitors.^32−34^

Once a likely mechanism was identified for XO44, for which X-ray coordinates of the covalent adduct with EGFR are available,^25^ reaction energetics were computed also for the recently reported inhibitors UPR1444 and UPR1433. Minimum free-energy paths of EGFR sulfonylation were thoroughly analyzed and compared to identify the chemical determinants and key stereo-electronic factors at the basis of an efficient sulfonylation of EGFR catalytic lysine.

## Results and Discussion

### EGFR^L858R/T790M/C797S^–XO44 Reactant Complex Model

We started our investigation by preparing a Michaelis complex of EGFR^L858R/T790M/C797S^ and XO44 suitable for QM/MM mechanistic simulations by conveniently modifying the X-ray coordinates of the covalent adduct, in which EGFR Lys745 is sulfonylated by XO44 (see the Supporting Information, SI).^25^ During the preparation of the EGFR-XO44 noncovalent complex, special attention was given to the protonation state of Lys745.

**Figure 2:**
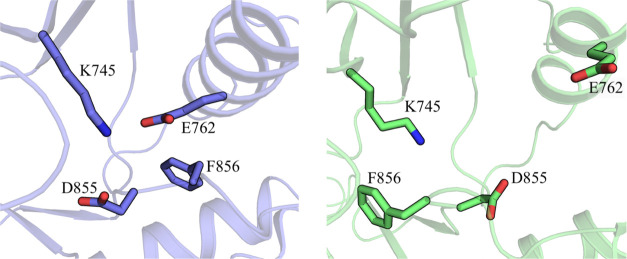
Lys745 environment in EGFR active state represented with violet carbon atoms (left, 1M17.pdb) and in EGFR inactive state colored with green carbon atoms (right, 5HG5.pdb).

According to structural data, XO44 recognizes an inactive state of EGFR, in which both the conserved Asp-Phe-Gly (DFG) motif and the αC-helix of the N-lobe of the kinase domain assume an “out” conformation.^35^ In this form, known as DFG-out/αC-out (DOCO) inactive state, the basicity of the catalytic lysine is significantly reduced (i.e., by nearly 2 p*K*_a_ units)^36^ compared to the DFG-in/αC-in (DICI) active state of the kinase. This p*K*_a_ downshift is due to the DICI/DOCO transition, which modifies the environment of Lys745. In the DICI state, the catalytic lysine forms salt bridges with Asp855 of the DFG motif and with Glu762 of αC-helix (Figure 2, left). Conversely, in the DOCO state, these interactions are missing since Lys745 is surrounded by hydrophobic residues, including Phe856 of the DFG motif (Figure 2, right). Calculations performed with the empirical scoring function PROPKA3 (which has been tested on 51 distinct lysine residues with an RMSE of 0.65 p*K*_a_ units)^37^ were consistent with the finding reported by Tsai and colleagues^36^ that, in the DOCO inactive state, the basicity of Lys745 is significantly reduced due to a perturbation of the environment. As a result, in the DOCO state, we estimated a downshift of the p*K*_a_ of nearly 2.6 log units, compared to the value estimated for the DICI state (predicted p*K*_a_ values = ∼9.0 (DOCO) vs 11.6 (DICI), Table S1).

The presence of a micro-environment, destabilizing the protonated form of Lys745 in the DOCO inactive state, along with kinetic data in solution showing that sulfonylation of N-acetyl lysine by aromatic sulfonyl fluorides is pH-dependent with the highest rate reached in basic conditions,^21^ indicates that Lys745 likely reacts with XO44 as a free base. Therefore, in the EGFR-XO44 Michaelis complex, the terminal amino group of Lys745 was modeled in its neutral form. The complex was equilibrated by classic molecular dynamics (MD) and, once the QM and MM regions were defined (Figure S1), a QM/MM MD simulation was performed. Then, the resulting structure was employed to evaluate likely mechanisms of sulfonylation by applying the adiabatic mapping (AM) approach.^38,39^

### Possible Mechanism of Lys745 Sulfonylation by XO44

**Scheme 1:**
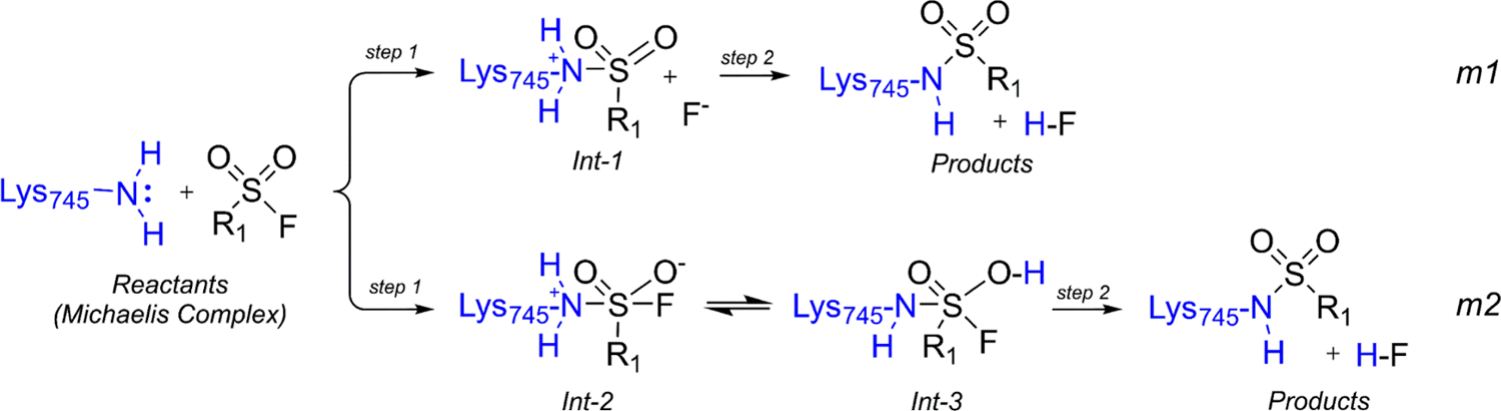
Alternative Mechanisms for Lys745 Sulfonylation by Aromatic Sulfonyl Fluorides, Based on What Have Been Previously Reported in Solution^40−42^

Nucleophilic substitution at the sulfonyl fluorides may occur by direct substitution of the fluorine atom (mechanism *m1*, Scheme 1)^40^ or by an elimination-addition pathway involving the generation of a trigonal bipyramidal intermediate (mechanism *m2*, Scheme 1).^41,42^

We started our investigation by simulating the first step of mechanism *m1* by means of an adiabatic mapping approach at the PM6/AMBER level,^43,44^ which emerged as a suitable method to investigate this kind of reactions, as suggested by simulations performed on cluster models using a set of convenient reaction coordinates (Figure S2 and Table S2).

**Figure 3:**
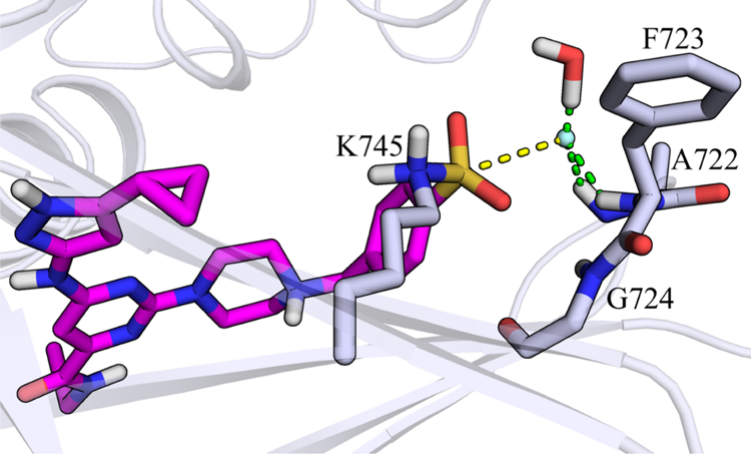
Structure of Int-1. Catalytic lysine Lys745 and p-loop residues (Ala722, Phe723, and Gly724) of EGFR interacting with the sulfonyl fluoride warhead of XO44 (**2**, pink carbon atoms) are represented with gray carbon atoms. The secondary structure of the kinase is displayed in gray cartoon. Polar interactions undertaken by XO44 with p-loop residues and a water molecule are highlighted as dashed lines colored in green. Yellow dashed lines represent the breakage of the S–F bond.

Step 1 was simulated reconstructing (i) a monodimensional (1D) potential energy surface (PES) using [d(S_SO2F_,F_SO2F_)-d(S_SO2F_,N_Lys745_)] as reaction coordinate (RC1), in which d(S_SO2F_,N_Lys745_) distance describes the nucleophilic attack at the sulfur center by Lys745 nitrogen, and d(S_SO2F_,F_SO2F_) distance accounts for the expulsion of the fluorine LG; (ii) a bidimensional (2D) PES in which d(S_SO2F_,N_Lys745_) and d(S_SO2F_,F_SO2F_) were explored as independent RCs (RC2 and RC3, respectively). Calculations showed that Int-1 was not a stationary point on both 1D and 2D PESs (Figure S3). Visual inspection of Int-1 geometry showed that the sulfonamide group displayed an arrangement resembling the one observed in the X-ray structure of EGFR-XO44 covalent adduct. The fluorine anion, while still close to the sulfur center, could form hydrogen-bond (H-bond) interactions with p-loop residues of EGFR active site (Figure 3). Despite these polar interactions, the high energy content of this configuration (∼28 kcal/mol over the level of the reactants) suggested that the enzymatic environment did not sufficiently stabilize the negative charge on the fluorine atom. This led us to speculate that sulfonylation of Lys745 does not occur following a direct substitution of the fluorine atom.

**Figure 4:**
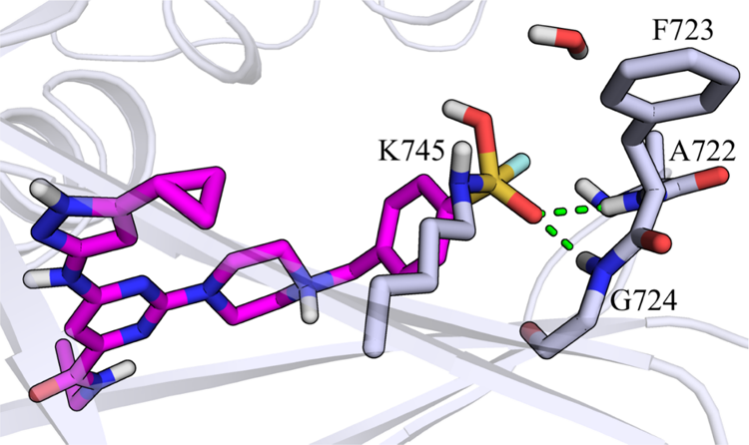
Structure of Int-3. Catalytic lysine Lys745 and p-loop residues (Ala722, Phe723, and Gly724) of EGFR interacting with the sulfonyl fluoride warhead of XO44 (**2**, pink carbon atoms) are represented with gray carbon atoms. The secondary structure of the kinase is displayed in gray cartoon. Green dashed lines represent polar interaction undertaken by XO44 with p-loop residues.

We continued our investigation simulating the first step of mechanism *m2* using two independent RCs. The first one, d(S_SO2F_,N_Lys745_) reported as RC2, accounting for the nucleophilic attack, and the second one (RC4), defined by the difference of two distances [d(N_Lys745_,H_Lys745_)-d(O_SO2F_,H_Lys745_)], describing the movement of one of the −NH_2_ hydrogens of Lys745 on the sulfonyl fluoride oxygen O_SO2F_ of XO44. Topology of the resulting 2D PES (Figure S4a) showed the presence of only two minima, corresponding to the geometries of the Michaelis complex and of the Int-3 intermediate (Figure 4). Int-2 was not a stationary point on the QM/MM surface and a simple unrestrained minimization was sufficient to conduct the system back to the reactants. The energy barrier separating the Michaelis complex and Int-3 was rather high (∼28 kcal/mol, Figure S4a), while the energy content of the reaction intermediate was fairly lower (∼16 kcal/mol, Figure S4a). Visual inspection of the resulting geometries confirmed that Int-3 was characterized by a trigonal bipyramidal arrangement (Figure 4).

One of the sulfonyl fluoride oxygens O_SO2F_ was engaged in a set of H-bonds with p-loop residues of the kinase. Furthermore, the former hydrogen of Lys745, now bound to the other sulfonyl oxygen of XO44, appeared well oriented to be transferred to the fluorine atom to generate the final product of the reaction.

The subsequent adiabatic mapping performed employing the combination of distances [d(S_SO2F_,F_SO2F_) + d(O_SO2F_,H_Lys745_) – d(F_SO2F_,H_Lys745_)] as RC (RC5, Figure S4b), which described the simultaneous expulsion of the fluorine atom and its direct protonation, showed that the generation of the products from Int-3 requires only 5.0 kcal/mol to occur (Figure S4b). Analysis of the geometries along the path showed that S–F breakage occured once the protonation at the fluorine atom is completed. Mechanism *m2* emerged as a sounding mechanism for Lys745 sulfonylation, although the first step featured a rather high energy barrier (Figure S5).

To search for an explanation for the higher barrier of step 1, the geometry of TS1, which resulted from the synchronous nucleophilic attack by Lys745 nitrogen at the sulfur center and by the proton transfer from Lys745 to O_SO2F_, was thoroughly analyzed. The N–H–O atoms involved in this proton transfer were poorly aligned forming an angle of ∼106° (far from the ideal value of 180°), somehow accounting for the high energy barrier computed for mechanism *m2*.

**Scheme 2:**
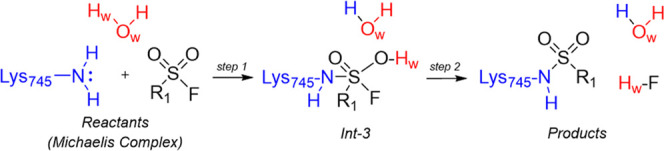
Mechanism *m3* for Lys745 Sulfonylation

We looked at the EGFR-XO44 X-ray adduct searching for residues that could assist the reaction. A crystallographic water molecule (*wat1*) was identified in proximity of Lys745 nitrogen, prompting us to hypothesize that such water molecule could act as proton shuttle for step 1. Calculations were thus repeated including this water molecule in the definition of the RCs, according to the mechanism *m3* reported in Scheme 2.

**Figure 5:**
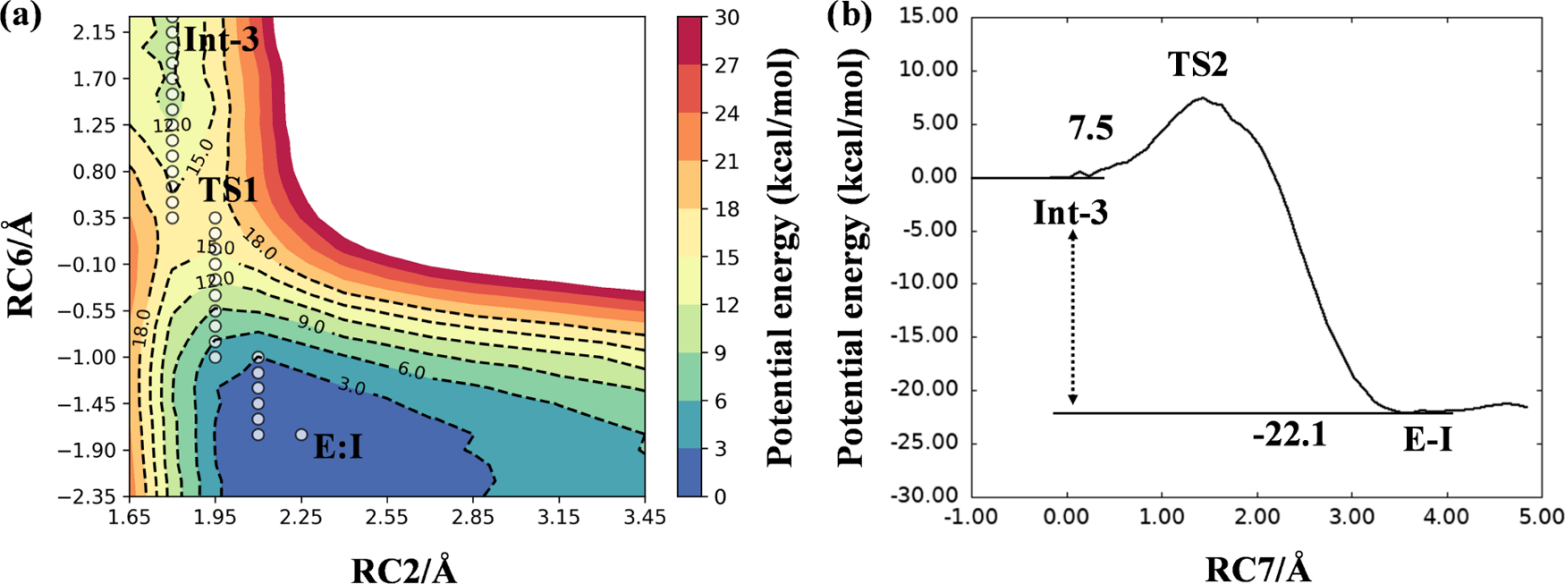
PM6/AMBER PESs for the inhibition mechanism *m3* by XO44. (a) Step 1 of the inhibition mechanism. RC2 corresponds to d(S_SO2F_,N_Lys745_). RC6 corresponds to [d(N_Lys745_,H_Lys745_) – d(O_w_,H_Lys745_) + d(O_w_,H_w_) – d(O_SO2F_,H_w_)]. White dots mark the minimum potential energy path. (b) Step 2 of the inhibition mechanism. RC7 corresponds to [d(S_SO2F_,F_SO2F_) + d(O_SO2F_,H_w_) – d(F_SO2F_,H_w_)].

The d(S_SO2F_,N_Lys745_) distance, accounting for the nucleophilic attack, and the polynomial expression [d(N_Lys745_,H_Lys745_) – d(O_w_,H_Lys745_) + d(O_w_,H_w_) – d(O_SO2F_,H_w_)], describing the movement of one of the −NH_2_ hydrogens of Lys745 on the oxygen atom of the water molecule O_w_ and the transfer of the one the hydrogens of the water molecule H_w_ on the oxygen atom O_SO2F_ of the sulfonyl fluoride warhead, were used as RCs (RC2 and RC6, respectively) to reconstruct a 2D PES (Figure 5a).

The 2D PES reported in Figure 5a showed that the first step of the inhibition mechanism *m3* of Lys745 sulfonylation took place with a significantly lower barrier (∼16 kcal/mol), consistent with a fast process of sulfonylation. The resulting intermediate Int-3 was employed to simulate step 2 using the combination of distances [d(S_SO2F_,F_SO2F_) + d(O_SO2F_,H_w_) – d(F_SO2F_,H_w_)] as RC. The 1D PES reported in Figure 5b revealed that LG expulsion was prompted by protonation of the fluorine atom, requiring to overcome a barrier of ∼7.5 kcal/mol to occur. The formation of a neutral product was also evidenced by a charge analysis performed on the reactive atoms showing that, differently from mechanism *m1*, the fluorine atom did not assume a net negative charge (Figures S6–S8).

**Figure 6:**
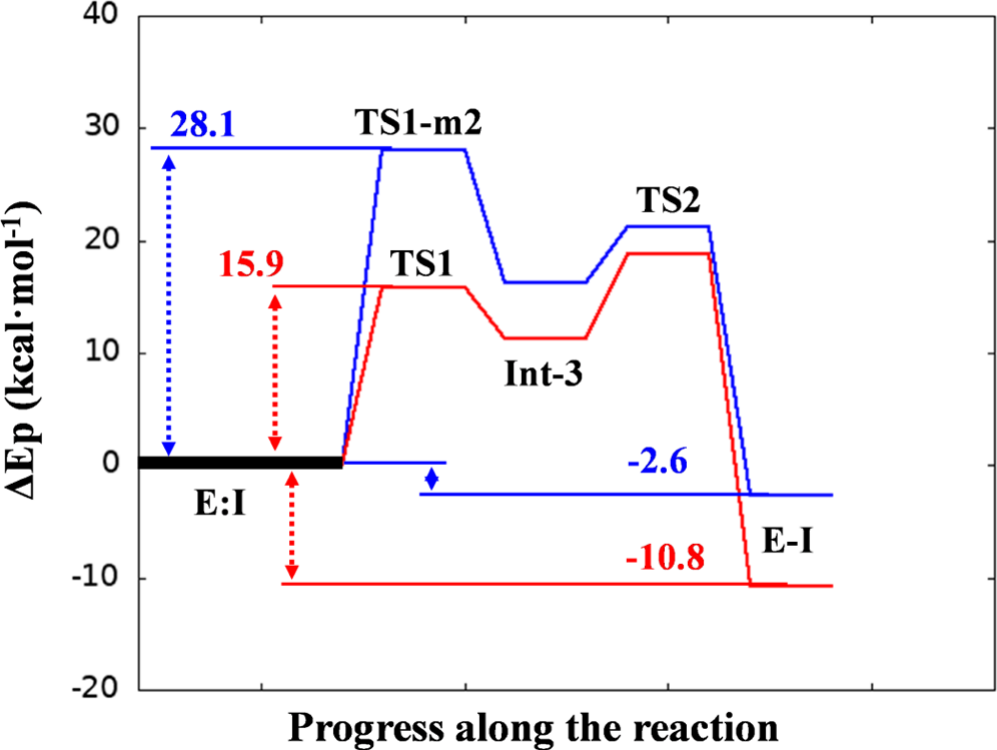
PM6/AMBER potential energy profile describing the inhibition mechanisms *m2* (blue line) and *m3* (red line) by XO44.

According to the identified minimum potential energy paths, mechanism *m3* (Figure 6, red line) emerged as the most likely mechanism for Lys745 sulfonylation by XO44 in EGFR. This result pointed out that the architecture of EGFR binding site favors, among reasonable sulfonylation mechanisms that may occur in solution,^40−42^ a specific process in which proton transfers assisted by a water molecule concur to lower the energy barriers of the key TSs of the SuFEx reaction.

### Potential of Mean Force (PMF) for Lys745 Sulfonylation

As the next step of our investigation, we employed umbrella sampling (US)^45^ simulations coupled to WHAM analysis^46,47^ to reconstruct the free-energy surface (FES) of Lys745 sulfonylation according to mechanism *m3*, which emerged as the one featured by the lowest potential energy barrier at PM6/AMBER level.^43,44^ The reaction was simulated in two steps, starting from geometries generated with the adiabatic mapping approach and equilibrated by QM/MM MD simulations (see the SI for details).

**Figure 7:**
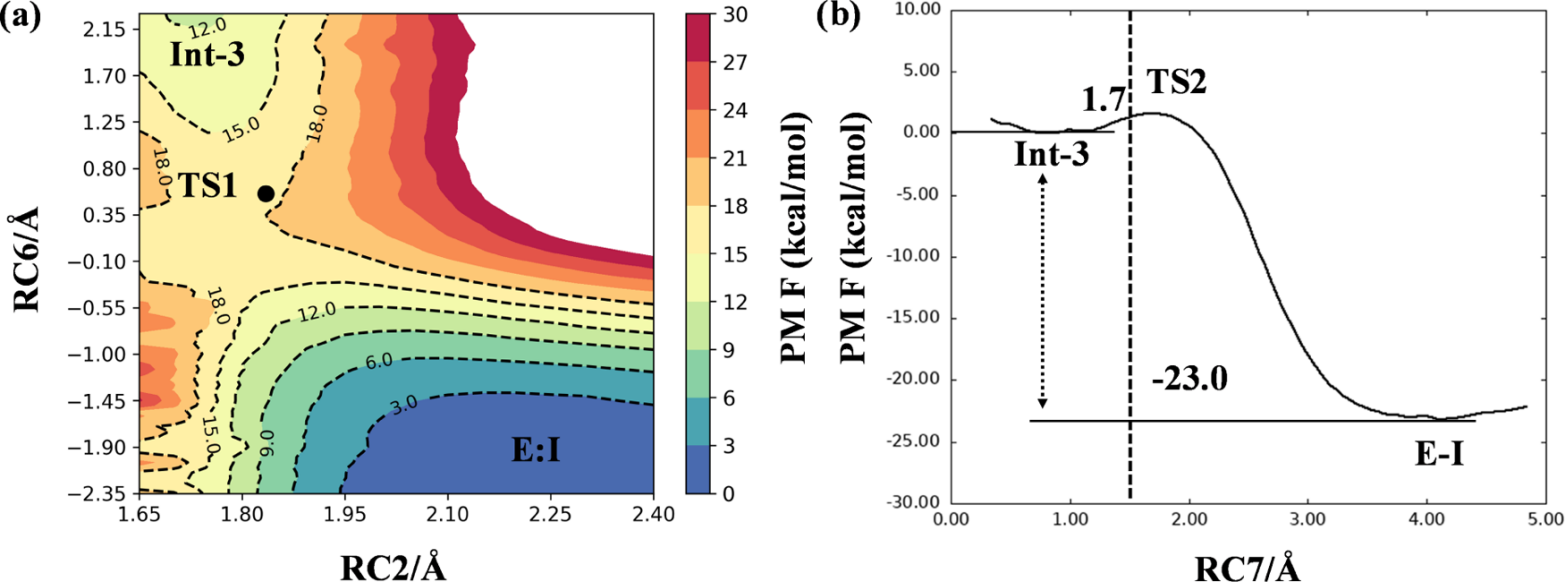
PM6/AMBER FESs for the inhibition mechanism *m3* by XO44. (a) Step 1 of the inhibition mechanism. RC2 corresponds to d(S_SO2F_,N_Lys745_) distance, and RC6 corresponds to the polynomial expression [d(N_Lys745_,H_Lys745_) – d(O_w_,H_Lys745_) + d(O_w_,H_w_) – d(O_SO2F_,H_w_)]. (b) Step 2 of the inhibition mechanism. RC7 corresponds to [d(S_SO2F_,F_SO2F_) + d(O_SO2F_,H_w_) – d(F_SO2F_,H_w_)]. The position of the optimized TSs at the M06-2X:6-31+G(d,p)/AMBER level is indicated as a black dot (a) and a dashed vertical line (b). Convergence was checked by assessing the evolution of free-energy profiles for each US window (Figure S9).

The FES of step 1 (formation of Int-3), reported in Figure 7a, was to some extent similar to the corresponding PES (Figure 5a). The minimum free-energy path connecting reactants and the trigonal bipyramidal intermediate Int-3 indicated that the presence of a concerted process in which nucleophilic attack and protonation of the O_SO2F_, via the crystallographic water molecule *wat1*, were tightly coupled events. The barrier for this chemical process was ∼17 kcal/mol, while the energy content of the newly formed intermediate was ∼11 kcal/mol.

**Figure 8:**
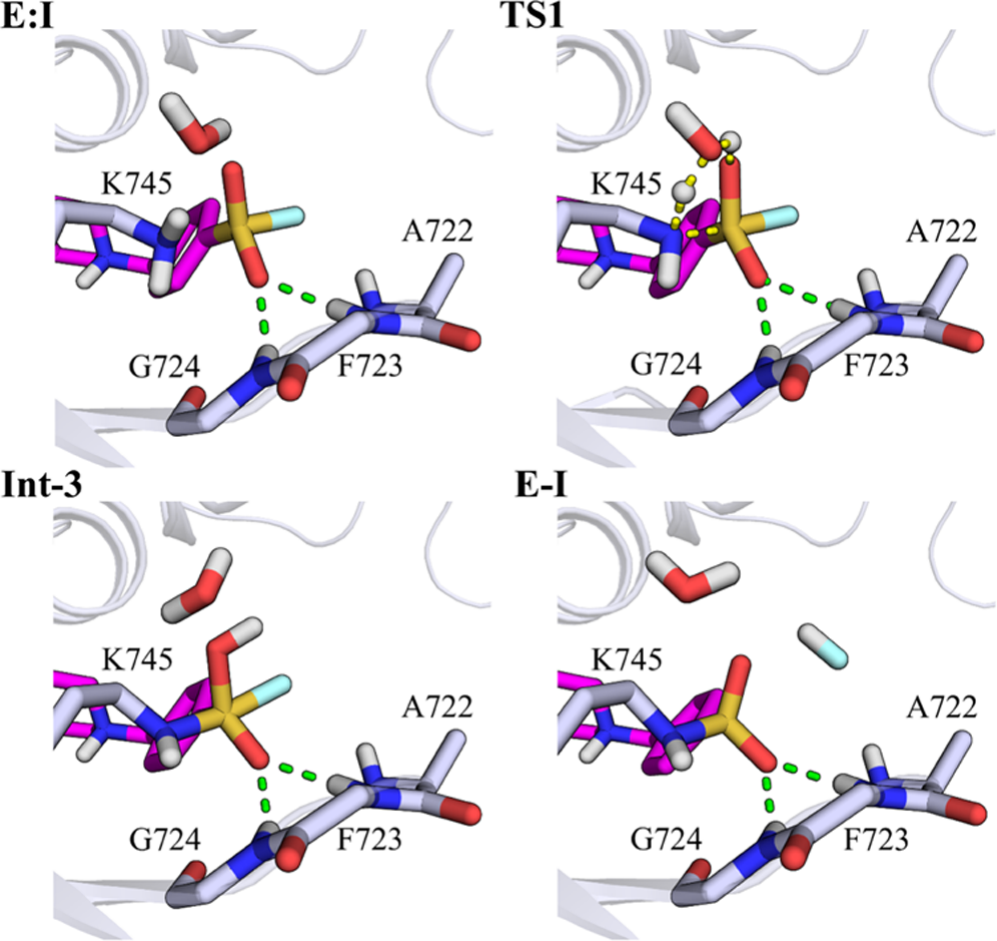
Representative snapshots of the key states of the proposed inhibition mechanism by XO44. Catalytic lysine Lys745 and p-loop residues of EGFR interacting with the sulfonyl fluoride warhead of XO44 (**2**, pink carbon atoms) are represented with gray carbon atoms. The secondary structure of the kinase is displayed in gray cartoon. Green dashed lines represent polar interactions undertaken by XO44 with p-loop residues, and yellow dashed lines underline bond forming and breaking.

Analysis of configurations along the minimum free-energy path showed that the transition state region TS1 was populated by geometries resembling six-membered-ring structures, with the water oxygen of *wat1* (O_w_) involved in two proton transfer reactions and the sulfur center at the two opposite endings of the “ring” (Figure 8, TS1 configuration; Figure S10). The FES of the second step showed the presence of a nearly spontaneous process (Figure 7b). These structures were well stabilized by H-bond interactions involving the sulfonyl fluoride warhead and the −NH backbone groups of the p-loop (Figure 8), similarly to what observed in the preliminary exploration of the PES. LG protonation and its subsequent expulsion generated the final product of the reaction overcoming a free-energy barrier lower than ∼1.7 kcal/mol. The second step was highly exergonic with the reaction product significantly more stable than the reactant by ∼11 kcal/mol. With an activation energy (*E*_act_) of ∼17 kcal/mol, formation of Int-3 emerged as the rate-limiting step of the whole reaction. This barrier was consistent with experimental data on kinase domain modification, showing that catalytic lysine sulfonylation by XO44 is a rapid process (estimated barrier significantly lower than 20 kcal/mol).^48^

TS structures, optimized at the M06-2X:6-31+G(d,p)/AMBER level, laid in the quadratic region of the PM6/AMBER FESs (Figure 7). Subsequent IRC calculations from optimized TSs led to geometries consistent with E:I, Int-3, and E-I minima, confirming the reaction path deduced from the PM6/AMBER FESs (see the Methods section for details).

Unrestrained QM/MM MD simulations of Int-3 showed that in the time scale of hundreds of picoseconds, this intermediate spontaneously converted in the reaction product, confirming that Int-3 was a transient configuration along the path.

### Path Collective Variables (PCVs) Simulations

To further support mechanism *m3* while describing the energetics of the overall reaction without separating it into discrete chemical steps (i.e., step 1, from reactants to Int-3, and step 2, from Int-3 to the products), we applied the path collective variables (PCVs) approach.^49 −52^ This method allowed to connect reactants and products in a single simulation exploring the possible concerted nature of chemical reactions.

The PCVs method requires the definition of solely two descriptors, representing the progress along a reference reaction path (*S*), and the distance from it (*Z*; see the Methods section for details). Geometries corresponding to configurations connecting the reactants to Int-3 and Int-3 to the products (Figure 5) were used to build an initial guess-path that was optimized through a set of consecutive QM/MM steered-MD (SMD) simulations.^53^ The variable *S* assured the progress of the reaction starting from the reactants (*S* = 0) up to the formation of the products (*S* = 1), and the variable *Z* allowed the system to explore geometries alternative to those defining the guess-path. Analysis of the geometries collected from consecutive SMD simulations (Figures S11 and S12) showed that the nodes of the reference path were minorly affected by the optimization procedure, suggesting that the starting guess-path was constituted by low-energy configurations. The optimization was thus stopped after six consecutive runs, and we performed five independent SMD simulations on the converged path to obtain information on reaction energetics.

**Figure 9:**
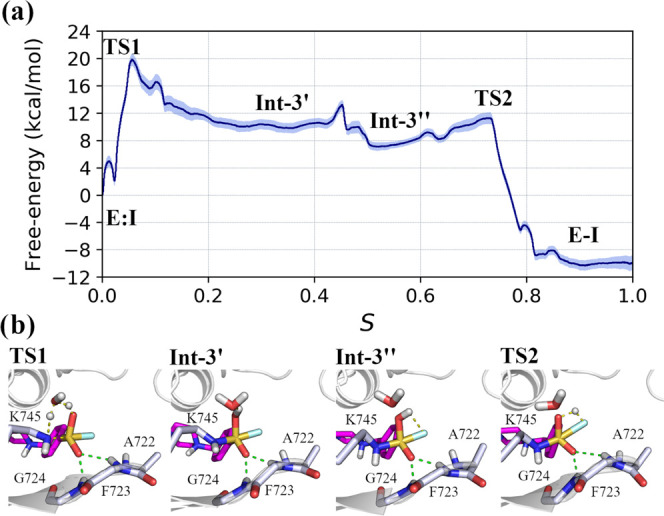
Lys745 sulfonylation by XO44. (a) Averaged free-energy profile of the reaction from reactants (E:I) to products (E-I); light-blue shadow represents the standard deviation. (b) Geometries of relevant steps (TS1 to TS2), taken from one of the five SMD simulations performed on the converged path, are represented. Catalytic lysine Lys745 and p-loop residues of EGFR interacting with the sulfonyl fluoride warhead of XO44 (**2**, pink carbon atoms) are represented with gray carbon atoms. The secondary structure of the kinase is displayed in gray cartoon. Green dashed lines represent polar interactions undertaken by XO44 with p-loop residues, and yellow dashed lines underline bond forming and breaking.

The resulting free-energy profile, calculated according to Jarzynski’s equality,^54,55^ and the geometries of relevant configurations are reported in Figure 9. Consistently with simulations based on the US with classical RCs, the reaction initiated with the concerted nucleophilic attack by Lys745 nitrogen atom at the sulfur center, and the proton transfer from Lys745 to *wat1* and from *wat1* to O_SO2F_ (Figure 9b, TS1). Configurations of the system corresponding to this event featured the same geometries that populated the TS1 region identified by US simulations, with the peculiar six-membered ring structure in which the oxygen atom of *wat1* and the sulfur center were placed at the opposite ends of the ring (Figure S10). The barrier of this event was of ∼20 kcal/mol (a value 3 kcal/mol higher compared to the one from US simulations) and led to the formation of a metastable intermediate (Int-3′) with an energy content of ∼11 kcal/mol, that evolved to a lower-energy intermediate (Int-3″, ∼8 kcal/mol) in which the O_SO2F_H_w_ group attached to sulfur center pointed toward the F_SO2F_ atom. This intermediate rapidly progressed to the formation of the product of the reaction, passing an energy barrier of only ∼4 kcal/mol in which protonation and expulsion of the LG occured (TS2). In accordance with the results of US simulations, the water-assisted nucleophilic attack performed by Lys745 thus emerged as the rate-limiting step of the entire reaction.

### Reaction Energetics for Other Sulfonyl Fluoride Derivatives

As a final step of our investigation, we reconstructed the FESs of Lys745 sulfonylation also for UPR1444 and UPR1433 inhibitors. To this end, we applied the same supervised computational protocol, initially employed for XO44, which, being based on reaction coordinates coupled with US simulations, allowed a finer description of the reaction energetics.^56^ Analysis of the resulting PESs and FESs showed that the three inhibitors share the same mechanism (Figures S13–S18) with the formation of Int-3 as the rate-limiting step of the overall process.

**Figure 10:**
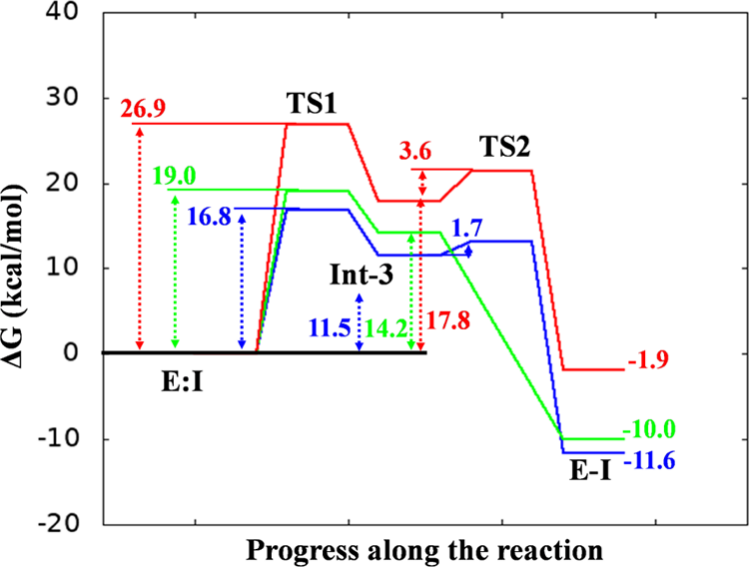
PM6/AMBER free-energy profiles derived from the PMFs for the inhibition mechanism of EGFR by XO44 (blue line), UPR1444 (green line), and UPR1433 (red line).

Activation energy barriers (*E*_act_) for XO44, UPR1444, and UPR1433 (∼17, ∼19, and ∼27 kcal/mol, respectively, Figure 10) were qualitatively consistent with inhibition potencies reported for these compounds (6, 1.6, and 146 nM, respectively).^26^ Calculations also provided a mechanistic rationale for the different kinetic behaviors displayed by these inhibitors. While XO44 and UPR1444 efficiently sulfonylated Lys745, leading to a stable covalent adduct, UPR1433 failed to covalently engage the same lysine when tested in similar experimental conditions.^26^ Moreover, the reaction-free energies (calculated as the difference between the energies of the product and the reactant) indicated that EGFR sulfonylation by both XO44 and UPR1444 was highly exergonic, with a computed Δ*G* lower than or equal to −10 kcal/mol (Figure 10). This computational finding agreed with the irreversible mechanism of action displayed by both UPR1444 and XO44. Recent findings on sulfur(VI) fluoride agents suggest that the accessibility of the lowest unoccupied molecular orbital (LUMO) controls the reaction rate of the SuFEx process.^48^ This seems to not be the case for the class of aromatic sulfonyl fluorides considered here. UPR1433, while possessing a LUMO energy value significantly lower than that of XO44 and UPR1444 (Table S3), resulted less efficient in the sulfonylation of Lys745. This suggests that the enzymatic environment is at least as important as the intrinsic reactivity of the warhead in the context of aliphatic amine sulfonylation by aromatic sulfonyl fluorides.

**Figure 11:**
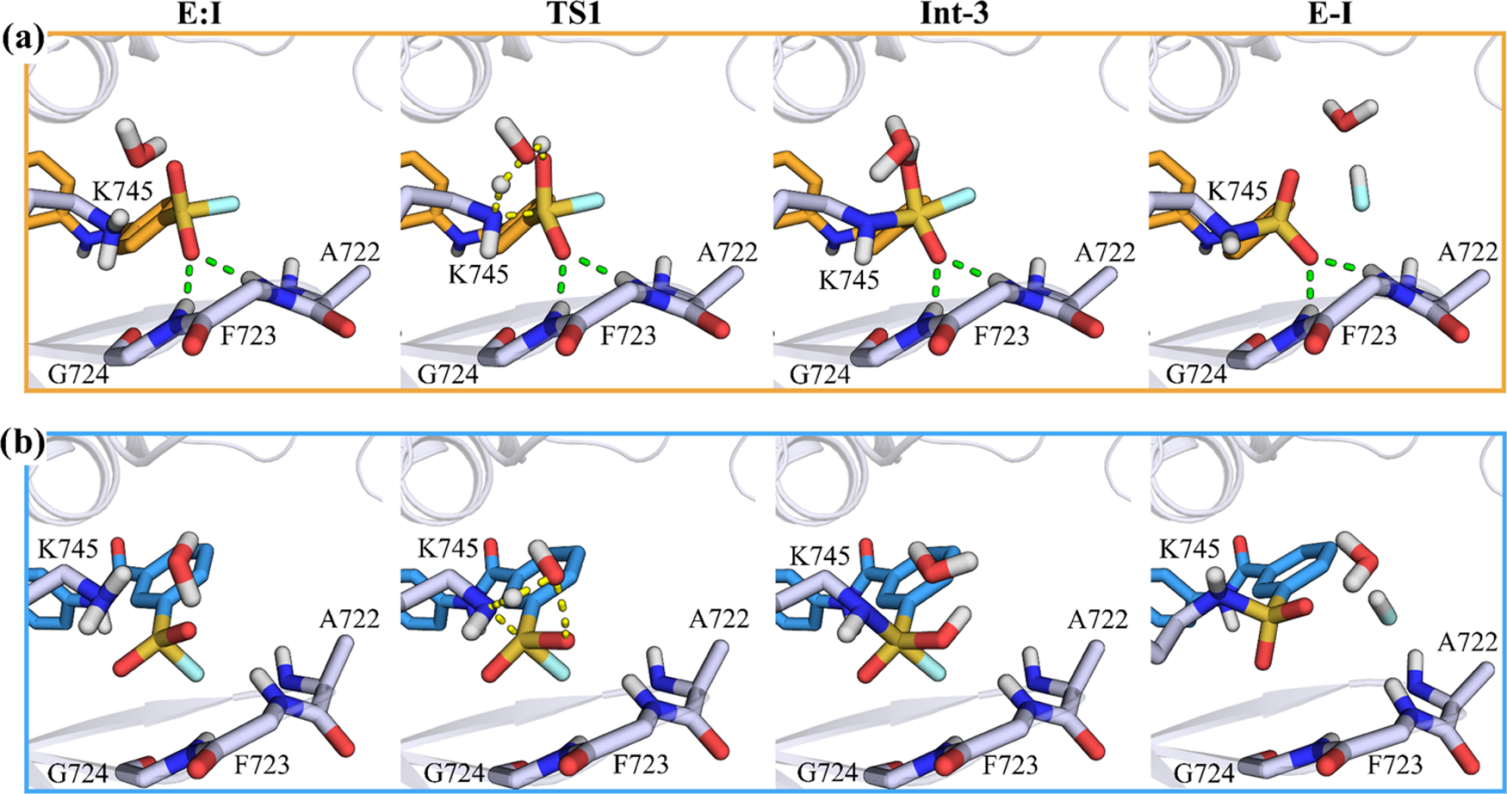
Representative snapshots of the key states of the proposed inhibition mechanism by UPR1444 (**3**, orange carbon atoms, a) and UPR1433 (**4**, blue carbon atoms, b). Catalytic lysine Lys745 and p-loop residues of EGFR interacting with the sulfonyl fluoride warhead of UPR1444/UPR1433 are represented with gray carbon atoms. The secondary structure of the kinase is displayed in gray cartoon. Green dashed lines represent polar interactions undertaken by UPR1444 with p-loop residues, and yellow dashed lines underline bond forming and breaking.

We thus analyzed the geometries along the path connecting E:I and Int-3 for the three inhibitors to search for structural determinants accounting for the different computed *E*_act_. From this geometry analysis, the role of H-bond interactions, involving the sulfonyl fluoride warhead and the –NH backbone group of the p-loop residues, emerged. The average interatomic distances obtained from the PM6/AMBER FESs calculated for the first step of the reaction (Tables S4–S6) indicated that strong interactions were established between the O_SO2F_ atom, not involved in the proton transfer, and the p-loop residues Phe723 and Gly724 during the reaction of EGFR with both XO44 (O_SO2F_–H_Phe723_ distance 2.04 ± 0.16 Å and O_SO2F_–H_Gly724_ distance 2.16 ± 0.19 Å in the TS1, Table S4) and UPR1444 (O_SO2F_–H_Phe723_ distance 2.21 ± 0.21 Å and O_SO2F_–H_Gly724_ distance 3.06 ± 025 Å in the TS1, Table S5), as also shown in Figures 8 and 11a. These interactions were missing in the case of UPR1433 (O_SO2F_–H_Phe723_ distance 4.12 ± 0.22 Å and O_SO2F_–H_Gly724_ distance 3.32 ± 0.30 Å in the TS1, Table S6), which instead placed the fluorine atom under the p-loop (Table S6 and Figure 11b). The different arrangement assumed by the sulfonyl fluoride warhead of UPR1433 within the active site of EGFR^L858R/T790M/C797S^ can be attributed to the rigid carbamoyl linker connecting the recognition scaffold targeting the hinge region of the kinase and the warhead itself. In the case of XO44 and UPR1444, the presence of a flexible methylamino linker allowed to place the warhead in a region of the EGFR active site suitable for the attack by Lys745. Overall, these results suggest that the high energy of TS1 for UPR1433 is related to the lack of electrostatic stabilization provided by the environment.

## Conclusions

Even though the third-generation inhibitor osimertinib represents a valid therapeutic option for NSCLC patients, the occurrence of new mutations, including C797S, is responsible for the loss of effectiveness of osimertinib and other EGFR inhibitors in lung cancer therapies. For this reason, the search for inhibitors able to covalently bind the catalytic Lys745 has emerged as a promising approach to identify fourth-generation EGFR inhibitors for the treatment of NSCLC patients.

The application of atomistic investigations aimed at elucidating the catalytic mechanism of lysine sulfonylation is a key and unavoidable process to evaluate novel candidates targeting Lys745. In this work, we applied different hybrid QM/MM approaches using enhanced sampling methods, US and PCVs, to characterize the mechanism of EGFR^L858R/T790M/C797S^ sulfonylation by the reference sulfonyl fluoride agent XO44, and by two recently described inhibitors UPR1444 and UPR1433.

Analysis of reaction energetics and visual inspection of key geometries along the minimum free-energy path led to the identification of the key electrostatic interactions at the basis of an efficient sulfonylation of Lys745. Simulations indicated that the presence of stable protein dipoles in the active site (i.e., –NH backbone groups of Phe723 and Gly724), able to form hydrogen bonds with the sulfonyl fluoride warheads, is critical to efficiently stabilize the main TS of the reaction. This is consistent with recent findings reported by the Sharpless group showing that a “perfect electrostatic matchmaking” between S^VI^–F warhead and a protein environment is required to observe a sulfur fluoride exchange (SuFEx) process in enzymes.^22,23^

Our simulations pointed to the presence of a flexible linker connecting the S^VI^–F warhead to the hinge region binding scaffold, as the structural and chemical determinant allowing Lys745 modification. This is the case of XO44 and UPR1444 that, thanks to their methylamino linker, can accommodate their sulfonyl fluoride warhead close to the polar backbone of Phe723 and Gly724. On the contrary, UPR1433 bearing a rigid amide linker fails to accommodate the sulfonyl fluoride group in a position prone to form stabilizing interactions with Phe723 and Gly724 in the main TS (TS1) leading to Lys745 sulfonylation.

Overall, our computational approach was able to provide a sounding mechanism for sulfonylating agents and to discriminate irreversible from reversible inhibitors of EGFR^L858R/T790M/C797S^, thus emerging as a suitable protocol to evaluate novel potential TCIs targeting the catalytic lysine of EGFR.

## Methods

### Model Building and Equilibration

The Michaelis complexes of EGFR with **2**–**4** were prepared exploiting the covalent adduct of 5U8L.pdb X-ray structure (**2**) and previous covalent docking models (**3**–**4**).^26^ These covalent models were modified by restoring the sulfonyl fluoride warhead of the inhibitors, and the atom type of the terminal nitrogen of Lys745 was conveniently adjusted and modeled in its neutral form accordingly to PROPKA3 prediction (Table S1). The resulting Michaelis complexes were solvated, neutralized, and parametrized with AMBER.^44^ AMBER ff03 force field^57,58^ and GAFF^59^ were applied to model the protein and the inhibitors, respectively. The systems were further minimized with the AMBER ff03 force field and equilibrated under NVT and NPT conditions, increasing the temperature up to 300 K and gradually reducing constraints on both the inhibitor and the protein. The production phase was carried out for 10 ns under NVT conditions. Starting from these equilibrated structures, the reaction mechanisms were investigated using a QM/MM approach (see the Supporting Information, SI, for details).

### Application of the QM/MM Potential

We preliminary performed a computational test on semiempirical methods (AM1,^60^ PM3,^61^ and PM6^43^) available in AMBER aimed at identifying a reliable method to describe the reaction of sulfonylation of a primary amine by an aromatic sulfonyl fluoride compound. These results were compared with calculations performed by modeling the mechanism *m2* at M06-2X^62^ and MP2^63−65^ levels of theory with 6-31G+(d,p) as basis set^66^ employing the Gaussian16 program.^67^ Calculations on a cluster model point at PM6 as a fair approach to describe this process in terms of reaction energetics compared to calculations performed at M06-2X^62^ and MP2^63−65^ levels (see the SI for details).

For this reason, the QM region of EGFR–inhibitor complexes was described by the PM6 Hamiltonian^43^ while the rest of the system was described by the AMBER ff03 force field^57,58^ (Figure S1). Adiabatic mapping approach was applied for a preliminary evaluation of alternative reaction mechanisms for Lys745 sulfonylation (see the SI for details).

### US Simulation for Lys745 Sulfonylation

After the exploration of the different inhibition mechanisms by adiabatic mapping simulations at the PM6/AMBER level of theory, the free-energy surfaces of mechanism *m3* were reconstructed at the same level of theory by means of QM/MM US simulations.^45,68^ Potentials of mean force (PMFs) of every single step were obtained using the WHAM approach,^46,47^ including in the calculations only the production phase (60 ps) of each simulated window.

For Lys745 sulfonylation by XO44 192 and 51 windows of US simulations for the 2D and 1D FESs were required, respectively. In the case of UPR1444, 156 and 41 US windows were used to reconstruct the 2D and 1D FESs, respectively. For UPR1433, the 2D and 1D FESs were obtained by performing 228 and 49 windows of US simulations, respectively. For all of the inhibitors, each US window was simulated for 60 ps, for a total of 43 ns of simulations performed. Convergence of the computed PMFs is achieved for each US window with an estimated error on the computed energy lower than 0.5 kcal/mol (Figures S9, S15, and S18). Therefore, differences in *E*_act_ among the three compounds are statistically significant.

The minimum-energy paths on the surfaces (potential and free energy) were determined using MEPSA software.^69^

### Path Collective Variables (PCVs) Simulations

To generate the free-energy landscape of Lys745 sulfonylation by XO44, without dividing it into different chemical steps, we performed path collective variables (PCVs) simulations using the sander module of Amber16^70^ coupled with Plumed 2.4.1 (see the SI for details),^71,72^ as previously described.^56^

An initial guess-path was generated from representative geometries belonging to the PES of step 1 and step 2 of the proposed mechanism *m3* (taken from Figure 5), following the procedure described by Branduardi et al.^29^ The following atoms were used as reference atoms to describe the reaction path: (i) S_SO2F_, F_SO2F_, the two O_SO2F_ atoms, and the C atom at position 1 of XO44; (ii) the Cε, N_Lys745_, and the two H_Lys745_ of Lys745; and (iii) all of the atoms of the crystalized water molecule *wat1*. Forty frames, spaced by an average root-mean-squared deviation (RMSD) of 0.18 Å, represented the initial guess-path connecting the reactants to the products. Two descriptors, *S* and *Z*, were used to define the position of the reference atoms (*R*) with respect to the guess-path. The two variables are defined as
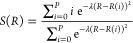
1
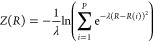
2where *i* is a discrete index ranging, in our study, from 0 to 1, (*R* – *R*(*i*))^2^ is the squared deviation from the *i*-th frame of the path, while *λ* is a smoothing parameter. The system was pulled along the variable *S* from 0 (Michaelis complex) to 1 (products) by applying a force constant of 300 kcal/mol, and the overall simulation was performed at a velocity of 0.3 *S* steps/ps. The variable *Z* was constrained by applying a quartic wall to allow the system to explore alternative geometries in proximity of the reference path while preventing it to move exceedingly far from the path. An upper limit was set to 0.005 Å^2^ with a force constant of 300 kcal/mol·Å^4^. At the end of each steered-MD (SMD) simulation,^53^ the frames collected were aligned to the first one and interpolated by applying the Catmull–Rom method,^73^ to generate a sufficiently large set of geometries from which to retrieve a smooth guess-path for a subsequent SMD/PCVs simulation. At each iteration, an RMSD-based distance matrix was computed to extract a new set of reference structures, equally separated from each other (∼0.01 Å), to be used as guess-path for the following SMD run. The procedure was applied iteratively for six rounds, when the reaction path did not change anymore moving from an SMD simulation to the following one (Figures S11 and S12). The converged path was finally used to perform five independent SMD/PCVs simulations. The final free-energy profile was obtained by averaging the work of the five simulations, following the application of Jarzynski’s equality in SMD simulations.^54,55^

### Characterization of the TSs at the DFT/AMBER Level of Theory

Structures of all important states involved in the reaction mechanisms (minima and transition state structures) were optimized at the M06-2X/6-31+G(d,p)/AMBER level,^62,66^ starting from representative PM6/AMBER snapshots from the FESs, with Gaussian 09^74^ coupled to the fDynamo library,^75,76^ as previously described (see the SI for details, Tables S7–S12).

## Data Availability

Molecular structures and input files used to run the simulations are made available as Supporting Information. The Amber16, Amber20, and AmberTools packages (https://ambermd.org) and Gaussian 09 (Revision A.01; https://gaussian.com) are available under license. PLUMED (https://www.plumed.org/) and fDynamo 2.2 (https://www.pdynamo.org/home) are open-source plugins.

## Supporting Information Available

Detailed description of the model building and equilibration procedure, technical details regarding the QM/MM simulations performed at the PM6/AMBER level and cluster model calculations; computational results and details for alternative mechanisms for sulfonylation by AM, US, and PCVs simulations; charge analysis of QM atoms for mechanisms *m1*–*m3*; details of characterization of the TSs at the DFT/AMBER level of theory; and frontier orbital energies for modeled inhibitors (PDF)
Geometries of the transition state (TS1) for EGFR sulfonylation by XO44, UPR1444, and UPR1433 (ZIP)
Input files of Path-CVs simulations (round 6; ZIP)

## Abbreviations

EGFR: epidermal growth factor receptor
NSCLC: non-small-cell lung cancer
TCI: targeted covalent inhibitors
SuFEx: sulfur fluoride exchange
QM/MM: quantum mechanics/molecular mechanics
RC: reaction coordinate
AM: adiabatic mapping
US: umprella sampling
PCVs: path collective variables
FES: free-energy surface
PES: potential energy surface
WHAM: weighted histogram analysis method
