# Supplementary material for: Mechanistic Modeling of Lys745 Sulfonylation in EGFR C797S Reveals Chemical Determinants for Inhibitor Activity and Discriminates Reversible from Irreversible Agents
Source: J Chem Inf Model. 2023 Feb 10;63(4):1301–12. doi: 10.1021/acs.jcim.2c01586 (PMC9976278; doi:10.1021/acs.jcim.2c01586)
Supplement: Supplementary file 1 — ci2c01586_si_001.pdf [file ci2c01586_si_001.pdf]

Supporting Information

Mechanistic Modeling of Lys745 Sulfonylation in EGFR C797S Reveals Chemical Determinants for Inhibitor Activity and Discriminates Reversible from Irreversible Agents.

*Kemel Arafet,[1,2] Laura Scalvini,[1] Francesca Galvani,[1] Sergio Martí,[2] Vicent Moliner,[2] Marco Mor,[1,3] and Alessio Lodola[1]**

[1] Dipartimento di Scienze degli Alimenti e del Farmaco, Università degli Studi di Parma, Parco Area delle Scienze 27/A, I-43124 Parma, Italy.

[2] BioComp Group, Institute of Advanced Materials (INAM), Universitat Jaume I, 12071 Castelló, Spain.

[3] Microbiome Research Hub, University of Parma, Parco Area delle Scienze 11/A, I-43124 Parma, Italy.

*E-mail: alessio.lodola@unipr.it

TABLE OF CONTENTS.

PROPKA PKA PREDICTION OF LYS745. 4

APPLICATION OF THE QM/MM POTENTIAL. 4

DEFINITION OF THE QM AND MM REGIONS FOR XO44, UPR1444, AND UPR1433. 4

CLUSTER MODEL CALCULATIONS. 5

QM/MM MODELING OF EGFR SULFONYLATION BY XO44. 8

POTENTIAL ENERGY SURFACES. 8

CHARGE ANALYSIS OF QM ATOMS FOR MECHANISMS *m1-m3*. 11

FREE ENERGY SURFACES. 13

IDENTIFICATION OF THE MINIMUM ENERGY PATH FOR EGFR SULFONYLATION BY XO44 ON PES. 15

ANALYSIS OF PATH COLLECTIVE VARIABLES (PCVS) SIMULATIONS. 16

QM/MM MODELING OF EGFR SULFONYLATION BY UPR1444. 18

POTENTIAL AND FREE ENERGY SURFACES 18

QM/MM MODELING OF EGFR SULFONYLATION BY UPR1433. 20

POTENTIAL AND FREE ENERGY SURFACES 20

FRONTIER ORBITAL ENERGIES FOR MODELED INHIBITORS. 22

ANALYSIS OF THE US GEOMETRIES CONNECTING E:I AND INT-3 FOR XO44, UPR1444, AND UPR1433. 23

CHARACTERIZATION OF THE TSS AT THE DFT/AMBER LEVEL OF THEORY. 25

REFERENCES. 30

Models building and equilibration.

The Michaelis complex of EGFR with XO44 was prepared starting from the EGFR-XO44 covalent adduct of the X-ray structure with PDB code 5U8L,[1] that was submitted to the Protein Preparation Wizard procedure.[2] The prepared EGFR-XO44 covalent model was geometrically optimized in gas phase with OPLS3e force field[3] to a root mean square displacement (RMSD) value of 0.3 Å.

The Michaelis complexes of EGFR with UPR1444 and UPR1433 were prepared exploiting the covalent adduct structures from previous covalent docking simulations, in which the Lys745 of the crystal structure PDB code 5U8L has been sulfonylated by these two inhibitors.[4]

All covalent models were modified by restoring the sulfonyl fluoride warhead of the inhibitors, and the atom type of the terminal nitrogen of Lys745 was conveniently adjusted and modelled in its neutral form. Michaelis complexes were subsequently submitted to a restrained geometric optimization with Macromodel[5] using OPLS3e force field.[3] The resulting Michaelis complexes were imported in t-leap for parametrization with AMBER.[6] AMBER ff03 force field[78] and general AMBER force field (GAFF)[9] were applied to model the protein and the inhibitors, respectively. A total of 4, 3, and 3 chlorine counter ions were added for the XO44, UPR1444 and UPR1433 models, respectively. Finally, the systems were solvated in orthorhombic boxes of TIP3P[10] water molecules (12155 for XO44, 15236 for UPR1444, and 15236 for UPR1433) with the following sizes: XO44 72.5 Å x 72.5 Å x 86.0 Å, UPR1444 73.0 Å x 91.4 Å x 82.0 Å, and UPR1433 73.0 Å x 91.3 Å x 82.2 Å. The systems were further minimized with the AMBER ff03 force field and equilibrated for 5 ns under NVT and 6 ns under NPT conditions, increasing the temperature up to 300 K and gradually reducing constraints on both the inhibitor and the protein. Hydrogen atoms were handled with the SHAKE algorithm, and a cut-off of 10 Å was selected to treat the electrostatic and van der Waals interactions. Long-range electrostatic interactions were treated using the particle mesh Ewald (PME) method. The production phase was carried out for 10 ns under NVT conditions.

PROPKA pK_a prediction of Lys745.

The protonation state of Lys745 was attributed accordingly to the prediction of the software PROPKA3.[11]

Table S1. Predicted pK_a values of Lys745 in different X-ray PDB codes.

X-ray PDB code	Predicted pK_a
1M17 (active state)	11.6
5HG5 (inactive state)	8.99

Application of the QM/MM potential.

Definition of the QM and MM regions for XO44, UPR1444, and UPR1433.

In this work, an additive hybrid QM/MM scheme was employed for the construction of the total Hamiltonian where the total energy is obtained from the sum of each contribution to the energy.

$$E_{QM/MM} = E_{QM} + E_{QM/MM}^{elect} + E_{QM/MM}^{vdW} + E_{MM} \qquad \text{(eq. 1)}$$

Here, E_{QM} describes the atoms in the QM region, $E_{QM/MM}$ defines the interaction between the QM and MM regions and E_{MM} describes the MM region. The QM region is described with the PM6 semiempirical Hamiltonian[12] and contains atoms of the inhibitor (17 for XO44, 19 for UPR1444, and 18 for UPR1433), 6 atoms of the residue Lys745, and a water molecule (Figure S1). The rest of the system was described by the AMBER ff03 force field.[7] Two hydrogen link atoms were added through the *qmmask* command of AMBER.[6]

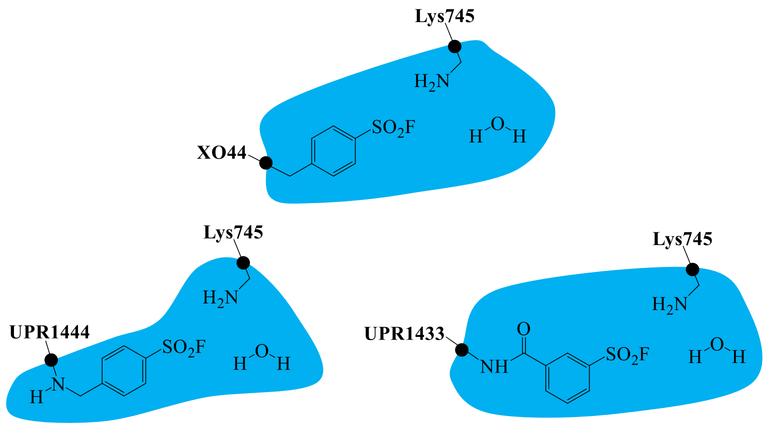

Figure S1. Details of the atoms of the active site treated quantum mechanically (blue region) in the computational models studied. Black dots represent the hydrogen link atoms.

Cluster model calculations.

We preliminary performed a computational test on some semiempirical methods available in AMBER aimed at identifying a reliable method to describe the reaction of sulfonylation of a primary amine by an aromatic sulfonyl fluoride compound. The primary amine and the aromatic sulfonyl fluoride compound are involved in the reaction, while the water molecules are using to simulate the interaction with the p-loop residues (Ala722, Phe723 and Gly724) of EGFR (Figure S2). All calculations were performed modeling the mechanism *m2* at AM1,[13] PM3,[14] PM6,[12] M06-2X[15]/6-31G+(d,p)[16] and MP2[17,18,19]/6-31G+(d,p)[16] levels of theory employing the Gaussian16 program.[20] Water solvation was simulated implicitly, using Cramer and Truhlar's SMD solvation model.[21]

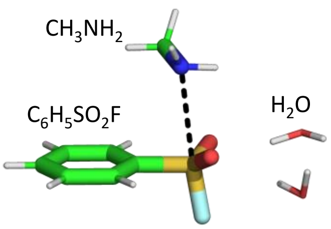

Figure S2. Details of the atoms of the cluster model. The primary amine and the aromatic sulfonyl fluoride compound are involved in the reaction, while the water molecules are using to simulate the interaction with the p-loop residues of EGFR.

The transition state geometries were optimized to a saddle point using an unconstrained transition state optimization. The resulting structures were characterized by frequency calculations, and the minimum energy path connecting reactants, intermediates and products through the transition state was evaluated by calculating the intrinsic reaction coordinate in both the forward and reverse directions to determine whether an intermediate could be found. The resulting potential energies of every single step, obtained for all levels of theory mentioned before, are shown in Table S2.

Table S2. Potential energies (kcal/mol) calculated at different levels of theory.

Potential Energy (kcal/mol)	AM1	PM3	PM6	M06-2X/6-31+G(d,p)	MP2/6-31G+(d,p)
$\Delta Ep_{(Int\text{-}2,E:I)}$	$\Delta Ep^{\neq}_{(TS1,E:I)} = 66.4$	17.9	-1.6	0.9	1.0
$\Delta Ep^{\neq}_{(TS1,Int\text{-}2)}$	$\Delta Ep_{(Int\text{-}3,E:I)} = 32.5$	31.4	29.5	36.0	37.1
$\Delta Ep_{(Int\text{-}3,Int\text{-}2)}$		2.7	16.5	25.6	28.9
$\Delta Ep^{\neq}_{(TS2,Int\text{-}3)}$	46.7	---	0.2	---	---
$\Delta Ep_{(E\text{-}I,Int\text{-}3)}$	-37.3	-32.4	-20.3	-41.1	-45.1

The first conclusion that can be derived from the analysis of Table S2 is that there are significant differences among results obtained by the methods employed. The AM1 method[13] was excluded due to the high activation potential energies (66.4 kcal/mol and 46.7 kcal/mol for the formation of Int-3 and E-I, respectively), especially during formation of E-I, generally characterized as a barrier-free process (see values of $\Delta Ep^{\neq}_{(TS2,Int\text{-}3)}$ on Table S2). The semiempirical methods PM3[14] and PM6[12] have different behaviors compared to the more accurate methods M06-2X[15] and MP2.[17,18,19] Although both semiempirical methods, PM3 and PM6, describe properly the activation potential energy of every single step, important differences are obtained comparing the stability of both Int-2 and Int-3 intermediates. PM6 method gives values of potential energy of the formation of both Int-2 (-1.6 kcal/mol) and Int-3 (16.5 kcal/mol) close to those obtained with M06-2X ($\Delta Ep_{(Int\text{-}2,E:I)}$=0.9 kcal/mol and $\Delta Ep_{(Int\text{-}3,Int\text{-}2)}$=25.6 kcal/mol) and MP2 methods ($\Delta Ep_{(Int\text{-}2,E:I)}$=1.0 kcal/mol and $\Delta Ep_{(Int\text{-}3,Int\text{-}2)}$=28.9 kcal/mol). On the contrary, the PM3 method gives worst values of potential energy for the formation of both Int-2 and Int-3 intermediates, with differences

greater than 15 kcal/mol for Int-2 and greater than 20 kcal/mol for Int-3 compared to both M06-2X and MP2 methods. Summarizing, PM6 is a fair approach to describe this process in term of reaction energetics when compared to calculations performed at M06-2X/6-31G+(d,p) and MP2/6-31G+(d,p) levels of theory.

QM/MM modeling of EGFR sulfonylation by XO44.

Potential energy surfaces.

Inhibition mechanism *m1* was explored by adiabatic mapping simulation at the PM6/AMBER level of theory (Figure S3).[12, 6] The study of the first step of the mechanism *m1* was carried out by both 1D and 2D PESs using different combinations of bond distances that describe the nucleophilic attack at the sulfur center by Lys745 nitrogen and the expulsion of the fluorine leaving group (LG).

A 1D PES (Figure S3a) was simulated using [$d(S_{SO2F},F_{SO2F})-d(S_{SO2F},N_{Lys745})$] as effective RC (RC1). The value of RC1 ranges from −1.90 to 1.00 Å, divided into 29 windows (step size 0.10 Å).

A 2D PES (Figure S3b) in which $d(S_{SO2F},N_{Lys745})$ and $d(S_{SO2F},F_{SO2F})$ were explored as independent reaction coordinates (RC2 and RC3, respectively) was also reconstructed. The value of RC2 ranges from 3.45 to 1.65 Å and RC3 ranges from 1.60 to 3.00 Å (step size of 0.10 Å). The 2D PES required 285 simulation windows.

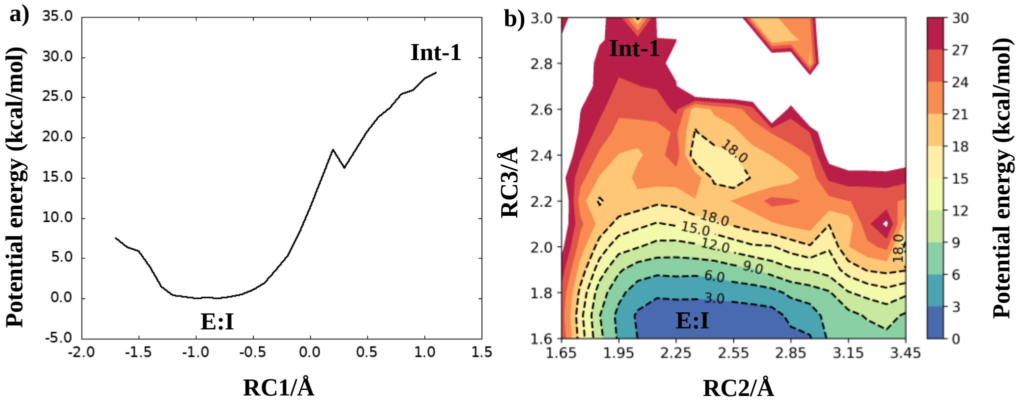

Figure S3. PM6/AMBER PESs for the inhibition mechanism *m1* by XO44. RC1 corresponds to [$d(S_{SO2F},F_{SO2F})-d(S_{SO2F},N_{Lys745})$]. RC2 corresponds to $d(S_{SO2F},N_{Lys745})$ and RC3 to $d(S_{SO2F},F_{SO2F})$.

Inhibition mechanism *m2* was explored by adiabatic mapping simulation at the PM6/AMBER level of theory (Figure S4).[12,6] The first step was simulated using two reaction coordinates (Figure S4a). The first one, RC2, describes the nucleophilic attack at the sulfur center by Lys745 nitrogen and is defined as $d(S_{SO2F},N_{Lys745})$. The second reaction coordinate, RC4, describes the proton

transfer from the Lys745 to the sulfonyl fluoride oxygen of XO44 and is defined as a combination of the following interatomic distances [d(N_{Lys745},H_{Lys745})-d(O_{SO2F},H_{Lys745})]. RC2 ranges from 3.45 to 1.65 Å (step size of 0.15 Å) and RC4 ranges from -1.70 to 1.70 Å (step size 0.10 Å). The 2D PES required 455 simulation windows. The second step was simulated as 1D PES using the combination of distances [d(S_{SO2F},F_{SO2F})+d(O_{SO2F},H_{Lys745})-d(F_{SO2F},H_{Lys745})] as RC5 (Figure S4b). RC5 describes the simultaneous expulsion of the fluorine atom and its direct protonation, and it ranges from 0.6 to 5.0 Å, with a step size of 0.10 Å. The 1D PES required 45 simulation windows.

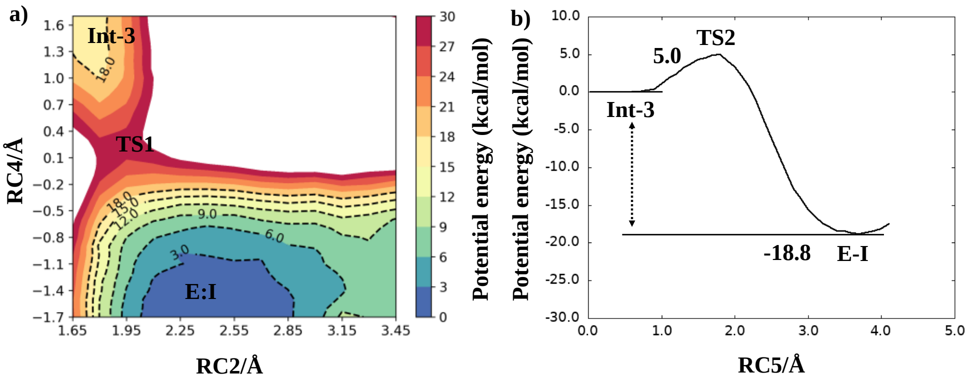

Figure S4. PM6/AMBER PESs for the inhibition mechanism *m2* by XO44. a) Step 1 of the inhibition mechanism. RC2 corresponds to d(S_{SO2F},N_{Lys745}). RC4 corresponds to [d(N_{Lys745},H_{Lys745})-d(O_{SO2F},H_{Lys745})]. b) Step 2 of the inhibition mechanism. RC5 corresponds to [d(S_{SO2F},F_{SO2F})+d(O_{SO2F},H_{Lys745})-d(F_{SO2F},H_{Lys745})].

Inhibition mechanism *m3* was initially explored by adiabatic mapping simulation at the PM6/AMBER level of theory.[12, 6] The first step was simulated using two reaction coordinates. The first one, RC2, describes the nucleophilic attack at the sulfur center by Lys745 nitrogen and is defined as d(S_{SO2F},N_{Lys745}). The second reaction coordinate, RC6, describes both proton transfer from the Lys745 to one of the oxygen atom of the water molecule and from the water molecule to the sulfonyl fluoride oxygen of XO44 and is defined as [d(N_{Lys745},H_{Lys745})-d(O_w,H_{Lys745})+d(O_w,H_w)-d(O_{SO2F},H_w)]. The value of RC2 ranges from 1.65 to 3.45 Å and RC6 ranges from -2.35 to 2.30 Å, with step size of 0.15 Å. The 2D PES required 416 simulation windows. The second step was simulated as 1D PES using the combination of distances

[$d(S_{SO2F},F_{SO2F})+d(O_{SO2F},H_w)-d(F_{SO2F},H_w)$] as RC7.. RC7 describes the simultaneous expulsion of the fluorine atom and its direct protonation, and it ranges from -0.16 to 4.84, with a step size of 0.10 Å. The 1D PES required 51 simulation windows.

Figure S5 reports the minimum potential energy path of mechanism *m2*, obtained with the MEPSA software.[22]

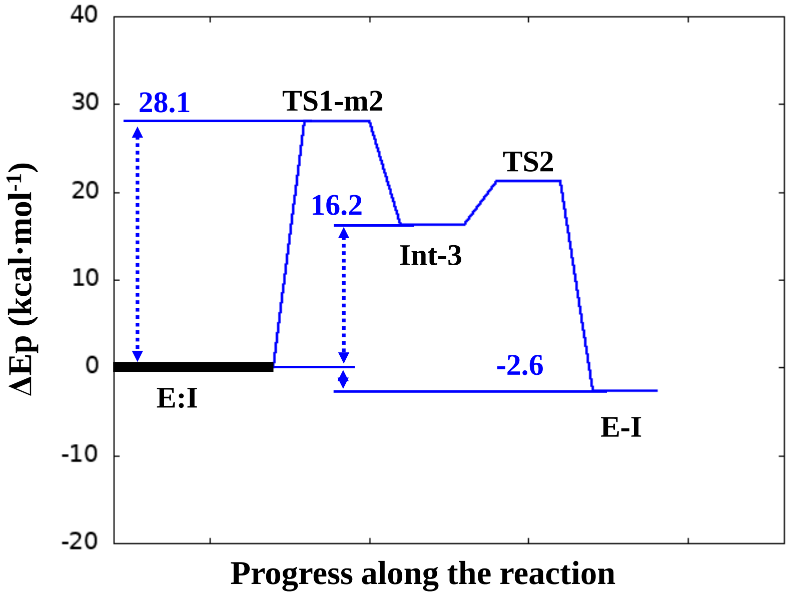

Figure S5. PM6/AMBER potential energy profile describing the inhibition mechanism *m2*.

Charge analysis of QM atoms for mechanisms *m1-m3*.

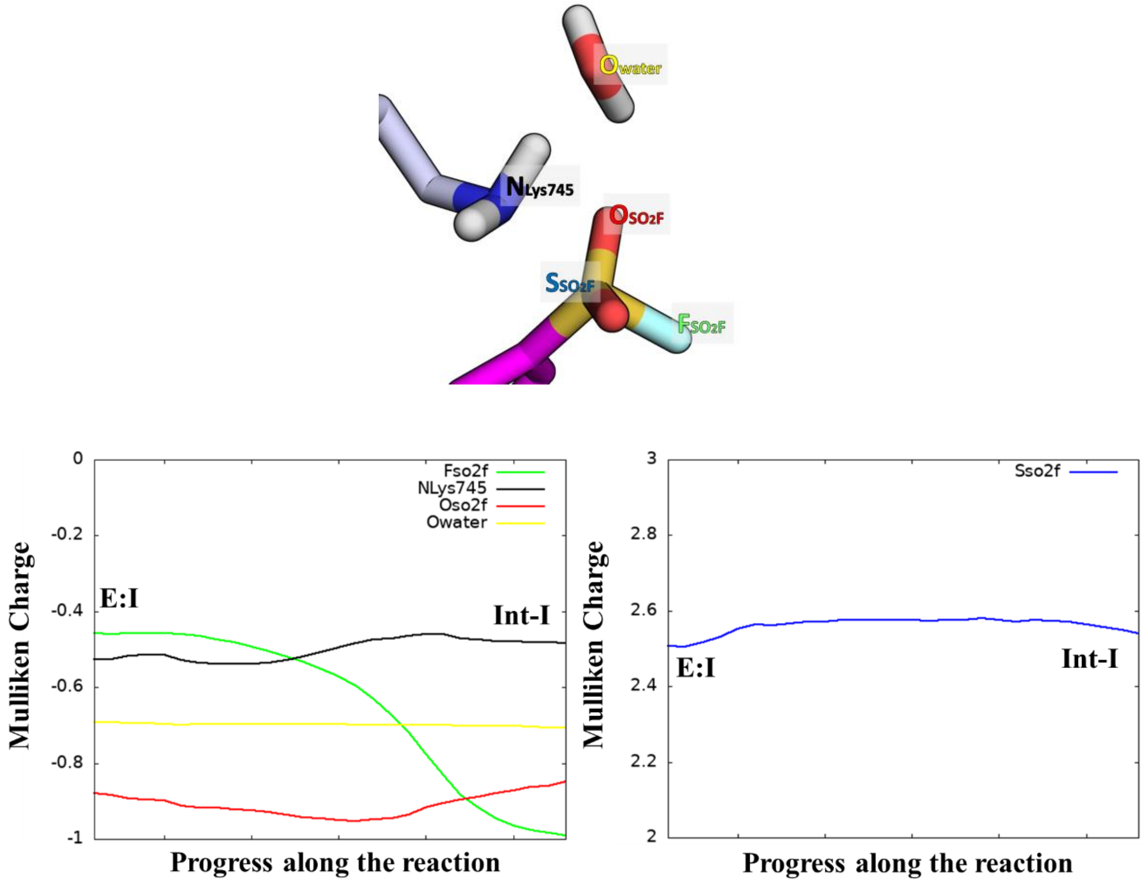

Figure S6. Evolution of the Mulliken charges of the key atoms along the inhibition mechanism *m1*.

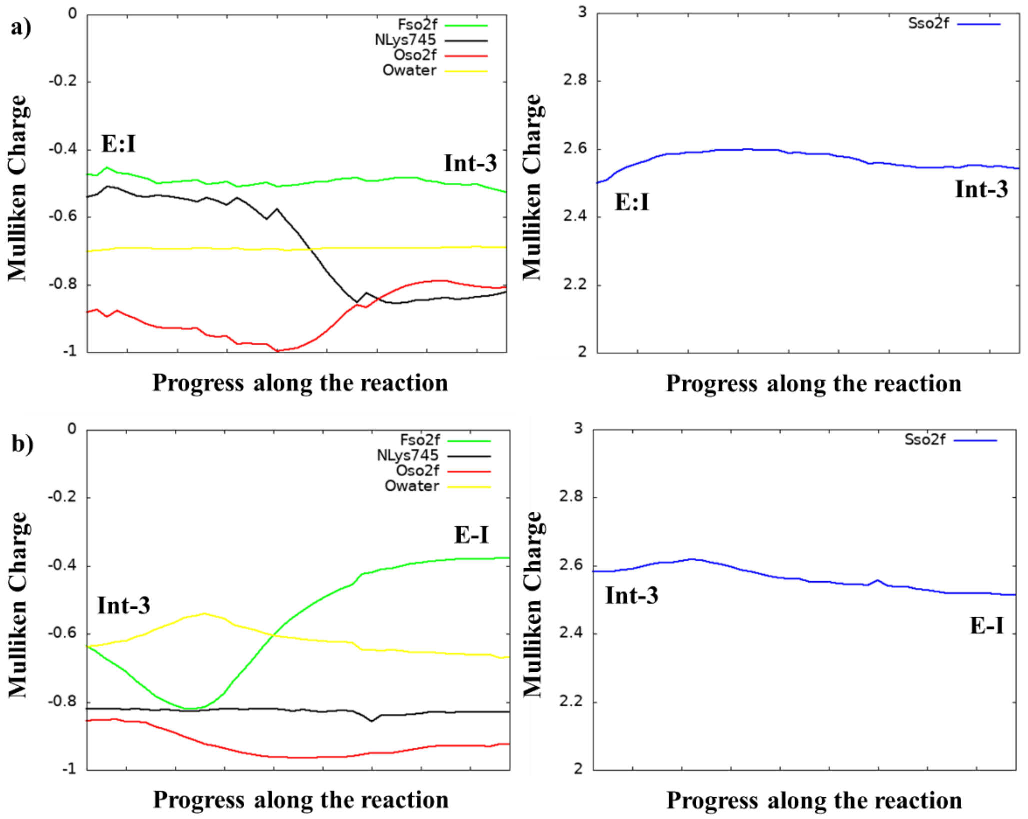

Figure S7. Evolution of the Mulliken charges of the key atoms along the minimum energy path of the inhibition mechanism *m2*. a) Step 1 of the inhibition mechanism. b) Step2 of the inhibition mechanism.

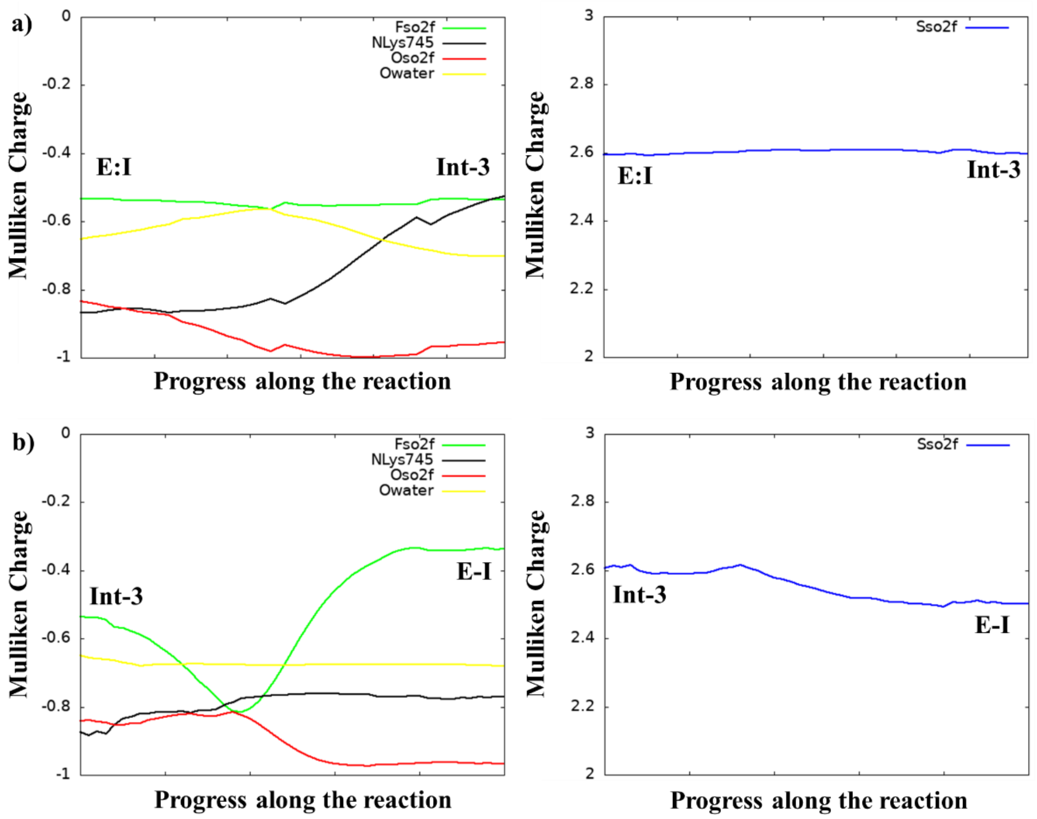

Figure S8. Evolution of the Mulliken charges of the key atoms along the minimum energy path of the inhibition mechanism *m3*. a) Step 1 of the inhibition mechanism. b) Step2 of the inhibition mechanism.

Free energy surfaces.

Once the 2D and 1D PESs corresponding to Lys745 sulfonylation by XO44 through the inhibition mechanism *m3* were obtained, the minimized structure obtained from the adiabatic mapping were used as a starting point for QM/MM umbrella sampling (US)[23] simulations. Regarding the 2D FES, the value of RC2 ranges from 1.65 to 2.40 Å, RC6 ranges from -2.35 to 2.30 Å, both with step size of 0.15 Å. In the case of the 1D FES, RC7 ranges from -0.16 to 4.84, with a step size of 0.10 Å. Harmonic restraints of 200 kcal·mol^{-1}·Å^{-2} were applied to the different reaction coordinates for the study of both steps of the inhibition mechanism *m3*. For each simulation window 13 ps of equilibration and 60 ps of production at 298 K were performed. Potentials of mean force (PMF) of every single step were obtained using weighted

histogram analysis method (WHAM) approach,[24,25] including in the calculations only the production phase of each simulated window.

Convergence of the computed PMFs is achieved for step 1 after 50 ps for each US window (Figure S9a) and for step 2 after 30 ps for each US window (Figure S9b).

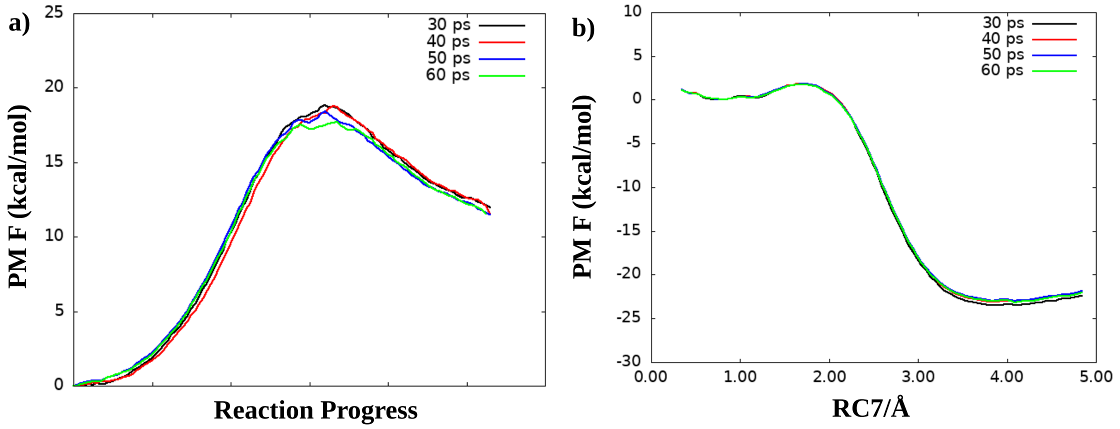

Figure S9. Evolution of the free-energy profiles at different simulation times for the inhibition mechanism *m3* by XO44. a) Step 1 of the inhibition mechanism. b) Step 2 of the inhibition mechanism.

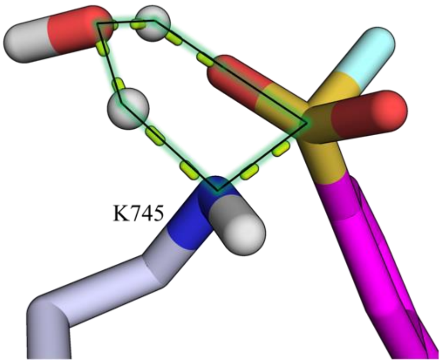

Figure S10. TS1 configuration for Lys745 sulfonylation by XO44. Green lines highlight the six-membered-ring structure with the water oxygen of *wat1* (O_w), involved in two proton transfer reactions, and the sulfur center (S_{SO2F}) at the two opposite ending of the “ring”.

Identification of the minimum energy path for EGFR sulfonylation by XO44 on PES.

The minimum energy path on the PES reported for EGFR sulfonylation by XO44 was determined using the minimum energy pathway analysis for energy landscapes (MEPSA) program.[22] Starting from a three-columns file reporting the energy values corresponding to each point of the map grid, the program samples the map and compares each point to the nearby points, checking if their value is equal, higher, or lower. This procedure performed on the whole map allows the identification of the local minima, called nodes. Following this step, the program finds the path connecting the origin point and the target point (corresponding to the reactants and the products, respectively) through an algorithm, named Global path generation method, that iteratively samples the map starting from the origin point and propagating toward the lowest energy points, until the target point is reached. The MEPSA records the iteration in which each point has been occupied, and once the target node is reached, a backward analysis from the target point to the origin is performed iteratively selecting the points with the lowest iteration number. This algorithm allows to identify the lowest energy path connecting nodes on the energy map.

Analysis of path collective variables (PCVs) simulations.

The analysis of path collective variables (PCVs) simulations revealed that geometries sampled during consecutive steered MD simulations (SMD) are minorly affected by the iterated optimization procedure, while a more significant reduction of the fluctuations of geometries around the guess path was registered from early SMD rounds to late ones. This behavior is evidenced in the More O'Ferral-Jencks (MOFJ) plots in which the combination of distances accounting for the nucleophilic attack/leaving group expulsion event and for the proton transfer from Lys745 to the crystalized water molecule *wat1* (Figure S11), and from *wat1* to the F_{SO2F} (Figure S12), are reported for a set of configurations from consecutive SMD rounds.

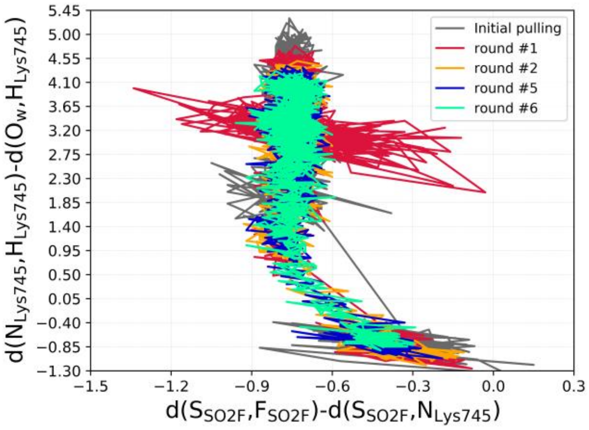

Figure S11. Exploration of the reaction path for Lys745 sulfonylation by XO44 from SMD/PCVs simulations. Evolution of the configurations in the space described by combination of distances describing the nucleophilic attack/leaving group expulsion [$d(S_{SO2F},F_{SO2F})$-$d(S_{SO2F},N_{Lys745})$] and proton transfer from Lys745 to the crystalized water molecule *wat1* [$d(N_{Lys745},H_{Lys745})$-$d(O_w,H_{Lys745})$]. Distances are expressed in Å.

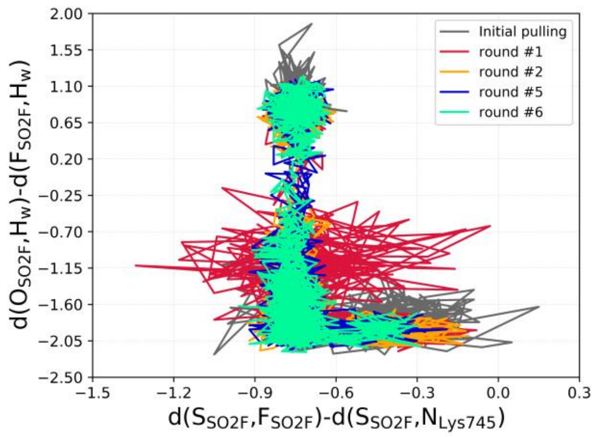

Figure S12. Exploration of the reaction path for Lys745 sulfonylation by XO44 from SMD/PCVs simulations. Evolution of the configurations in the space described by combination of distances describing the nucleophilic attack/leaving group expulsion [$d(S_{SO2F},F_{SO2F})$-$d(S_{SO2F},N_{Lys745})$] and proton transfer from the crystalized water molecule *wat1* to the F_{SO2F} atom [$d(O_{SO2F},H_w)$-$d(F_{SO2F},H_w)$]. Distances are expressed in Å.

QM/MM modeling of EGFR sulfonylation by UPR1444.

Potential and free energy surfaces.

Inhibition mechanism *m3* was initially explored by adiabatic mapping simulation at the PM6/AMBER level of theory (Figure S13).[12,6] Both inhibition steps were simulated using the same RCs as in the case of XO44. In the first step, RC2 corresponds to the nucleophilic attack $d(S_{SO2F},N_{Lys745})$ and RC6 is defined as $[d(N_{Lys745},H_{Lys745})-d(O_w,H_{Lys745})+d(O_w,H_w)-d(O_{SO2F},H_w)]$. The value of RC2 ranges from 1.65 to 3.45 Å and RC6 ranges from -1.90 to 1.85 Å, with a step size of 0.15 Å. The 2D PES required 338 simulation windows (Figure S13a). The second step was simulated as 1D PES using the combination of distances $[d(S_{SO2F},F_{SO2F})+d(O_{SO2F},H_w)-d(F_{SO2F},H_w)]$ as RC7 (Figure S13b). RC7 ranges from 1.07 to 5.07, with a step size of 0.10 Å. The 1D PES required 41 simulation windows.

Later, the minimized structure obtained from the adiabatic mapping were used as a starting point for QM/MM US simulations[23] (Figure S14). As for XO44, the value of RC2 ranges from 1.65 to 2.40 Å, RC6 ranges from -1.90 to 1.85 Å, both with step size of 0.15 Å. In the case of the 1D FES, RC7 ranges from 1.07 to 5.07, with a step size of 0.10 Å. Harmonic restraints of 200 $kcal \cdot mol^{-1} \cdot Å^{-2}$ were applied to the different reaction coordinates for the study of both steps of the inhibition mechanism *m3*. For each simulation window 13 ps of equilibration and 60 ps of production at 298 K were performed. PMFs of every single step were obtained using the WHAM approach,[24,25] including in the calculations only the production phase of each simulated window. Convergence of the computed PMFs is achieved after 30 ps for each US window for both inhibition steps (Figure S15).

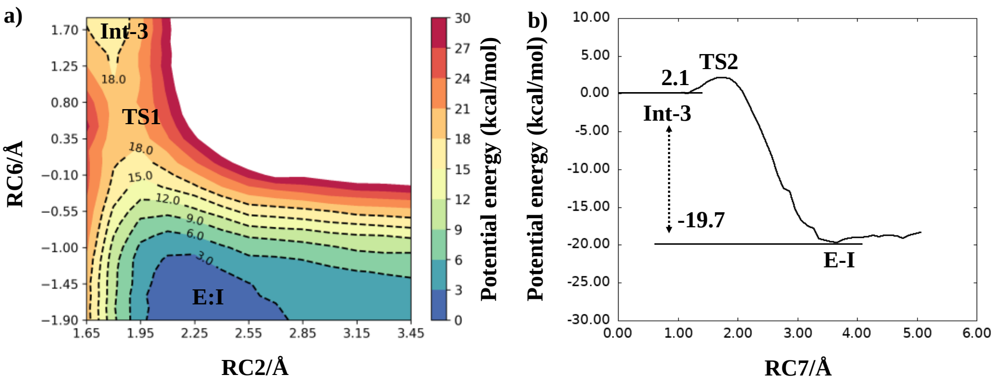

Figure S13. PM6/AMBER PESs for the inhibition mechanism *m3* by UPR1444. a) Step 1 of the inhibition mechanism. RC2 corresponds to $d(\mathrm{S_{SO2F}},\mathrm{N_{Lys745}})$. RC6 corresponds to $[d(\mathrm{N_{Lys745}},\mathrm{H_{Lys745}})-d(\mathrm{O_w},\mathrm{H_{Lys745}})+d(\mathrm{O_w},\mathrm{H_w})-d(\mathrm{O_{SO2F}},\mathrm{H_w})]$. b) Step 2 of the inhibition mechanism. RC7 corresponds to $[d(\mathrm{S_{SO2F}},\mathrm{F_{SO2F}})+d(\mathrm{O_{SO2F}},\mathrm{H_w})-d(\mathrm{F_{SO2F}},\mathrm{H_w})]$.

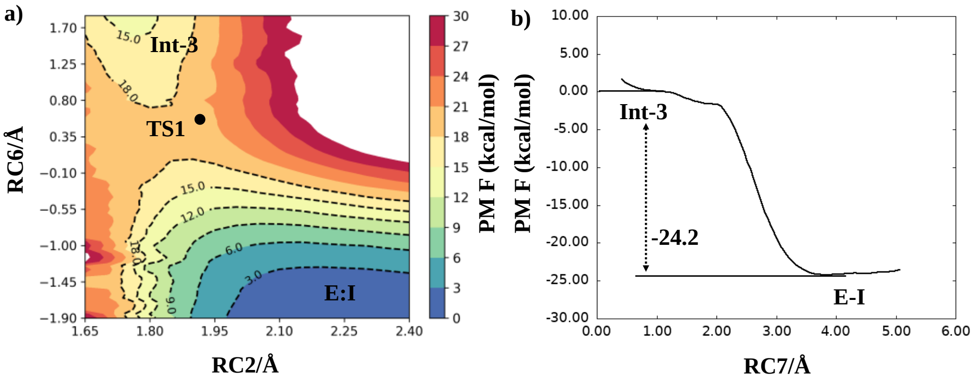

Figure S14. PM6/AMBER FESs for the inhibition mechanism m3 by UPR1444. a) Step 1 of the inhibition mechanism. RC2 corresponds to $d(\mathrm{S_{SO2F}},\mathrm{N_{Lys745}})$. RC6 corresponds to $[d(\mathrm{N_{Lys745}},\mathrm{H_{Lys745}})-d(\mathrm{O_w},\mathrm{H_{Lys745}})+d(\mathrm{O_w},\mathrm{H_w})-d(\mathrm{O_{SO2F}},\mathrm{H_w})]$. The position of the optimized TS1 at M06-2X:6-31+G(d,p)/AMBER level is indicated as black dot. b) Step 2 of the inhibition mechanism. RC7 corresponds to $[d(\mathrm{S_{SO2F}},\mathrm{F_{SO2F}})+d(\mathrm{O_{SO2F}},\mathrm{H_w})-d(\mathrm{F_{SO2F}},\mathrm{H_w})]$.

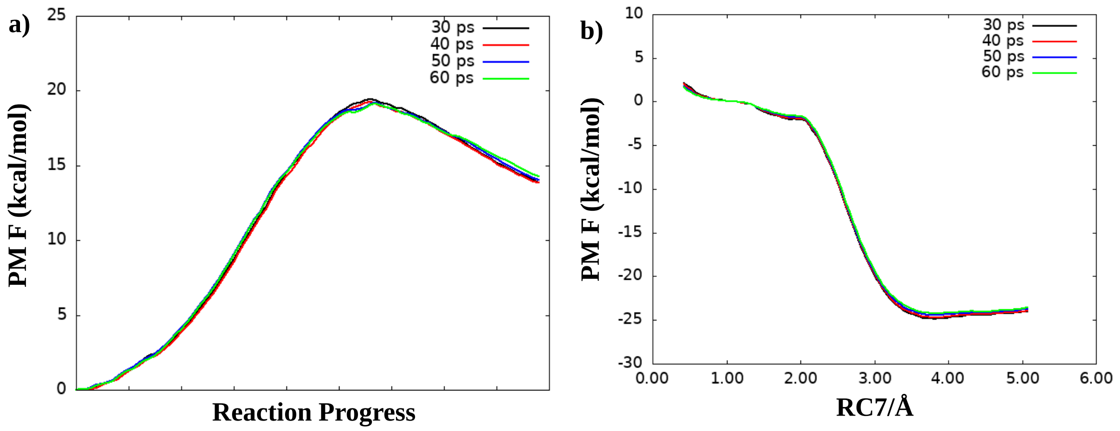

Figure S15. Evolution of the free-energy profiles at different simulation times for the inhibition mechanism *m3* by UPR1444. a) Step 1 of the inhibition mechanism. b) Step 2 of the inhibition mechanism.

QM/MM modeling of EGFR sulfonylation by UPR1433.

Potential and free energy surfaces.

Inhibition mechanism *m3* initially was explored by adiabatic mapping simulation at the PM6/AMBER level of theory (Figure S16).[12,6] Both inhibition steps were simulated using the same reaction coordinates as in the case of both XO44 and UPR1444. In the first step, RC2, is defined as $d(S_{SO2F},N_{Lys745})$ and RC6 corresponds to $[d(N_{Lys745},H_{Lys745})-d(O_w,H_{Lys745})+d(O_w,H_w)-d(O_{SO2F},H_w)]$. The value of RC2 ranges from 1.65 to 3.45 Å and RC6 ranges from -2.55 to 3.00 Å, with a step size of 0.15 Å. The 2D PES required 494 simulation windows (Figure S16a). The second step was simulated as 1D PES using the combination of distances $[d(S_{SO2F},F_{SO2F})+d(O_{SO2F},H_w)-d(F_{SO2F},H_w)]$ as RC7. RC7 ranges from 0.82 to 5.62, with a step size of 0.10 Å. The 1D PES required 49 simulation windows (Figure S16b).

Later, the minimized structure obtained from the adiabatic mapping were used as a starting point for QM/MM US[17] simulations (Figure S17). The value of RC2 ranges from 1.65 to 2.40 Å, RC6 ranges from -2.55 to 3.00 Å, both with step size of 0.15 Å. In the case of the 1D FES, RC7 ranges from 0.82 to 5.62, with a step size of 0.10 Å. Harmonic restraints of 200 $kcal·mol^{-1}·Å^{-2}$ were applied to the different reaction coordinates for the study of both steps of the inhibition

mechanism *m3*. For each simulation window 13 ps of equilibration and 60 ps of production at 298 K were performed. PMFs of every single step were obtained using WHAM approach,[24,25] including in the calculations only the production phase of each simulated window. Convergence of the computed PMFs is achieved after 30 ps for each US window for both inhibition steps (Figure S18).

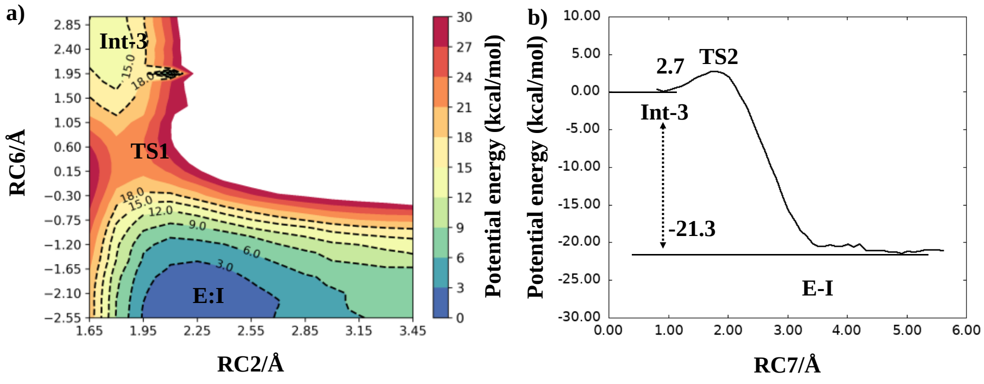

Figure S16. PM6/AMBER PESs for the inhibition mechanism *m3* by UPR1433. a) Step 1 of the inhibition mechanism. RC2 corresponds to $d(S_{SO2F},N_{Lys745})$. RC6 corresponds to $[d(N_{Lys745},H_{Lys745})-d(O_w,H_{Lys745})+d(O_w,H_w)-d(O_{SO2F},H_w)]$. b) Step 2 of the inhibition mechanism. RC7 corresponds to $[d(S_{SO2F},F_{SO2F})+d(O_{SO2F},H_w)-d(F_{SO2F},H_w)]$.

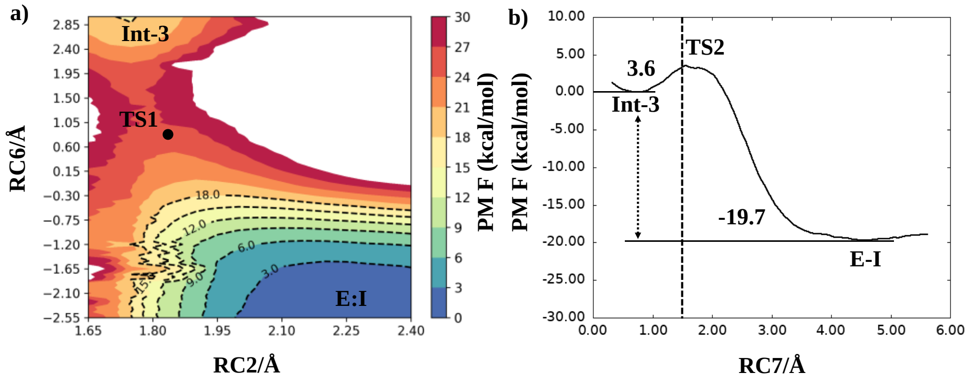

Figure S17. PM6/AMBER FESs for the inhibition mechanism *m3* by UPR1433. a) Step 1 of the inhibition mechanism. RC2 corresponds to $d(S_{SO2F},N_{Lys745})$. RC6 corresponds to $[d(N_{Lys745},H_{Lys745})-d(O_w,H_{Lys745})+d(O_w,H_w)-d(O_{SO2F},H_w)]$. b) Step 2 of the inhibition mechanism. RC7 corresponds to $[d(S_{SO2F},F_{SO2F})+d(O_{SO2F},H_w)-d(F_{SO2F},H_w)]$. The position of the optimized TSs at M06-2X:6-31+G(d,p)/AMBER level are indicated as black dot (a) and dashed vertical line (b).

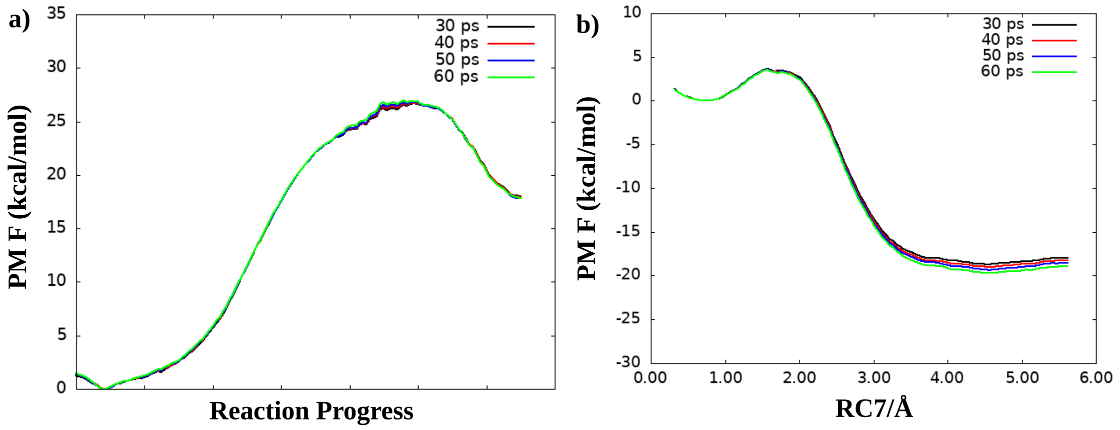

Figure S18. Evolution of the free-energy profiles at different simulation times for the inhibition mechanism *m3* by UPR1433. a) Step 1 of the inhibition mechanism. b) Step 2 of the inhibition mechanism.

Frontier orbital energies for modeled inhibitors.

Table S3. Frontier orbitals energies calculated in gas phase at the PM6 level of theory.

Frontier orbitals energies (Hartree)	XO44	UPR1444	UPR1433
E_{HOMO}	-0.26249	-0.28226	-0.28243
E_{LUMO}	-0.03465	-0.03897	-0.05512

Analysis of the US geometries connecting E:I and Int-3 for XO44, UPR1444, and UPR1433.

Table S4. Average distances (in Å) ± standard deviation for key states located along step 1 of the inhibition mechanism *m3* by XO44, obtained from the PM6/AMBER free energy surfaces.

Distances	E:I	TS1	Int-3
**Active Site**			
N_{Lys745} - S_{SO2F}	2.24 ± 0.05	1.84 ± 0.03	1.75 ± 0.03
F_{SO2F} - S_{SO2F}	1.68 ± 0.05	1.91 ± 0.10	1.81 ± 0.08
N_{Lys745} - H_{Lys745}	1.04 ± 0.03	1.52 ± 0.09	2.74 ± 0.19
O_w - H_{Lys745}	2.18 ± 0.03	1.16 ± 0.05	0.98 ± 0.04
O_w - H_w	0.97 ± 0.04	1.10 ± 0.04	1.62 ± 0.13
O_{SO2F} - H_w	2.20 ± 0.23	1.58 ± 0.10	1.06 ± 0.06
**SO_2F and p-loop residues**			
O_{SO2F} - H_{Ala722}	3.49 ± 0.30	2.95 ± 0.35	3.59 ± 0.35
O_{SO2F} - H_{Phe723}	2.09 ± 0.14	2.04 ± 0.13	2.13 ± 0.15
O_{SO2F} - H_{Gly724}	2.26 ± 0.23	2.16 ± 0.19	2.15 ± 0.21

Table S5. Average distances (in Å) ± standard deviation for key states located along step 1 of the inhibition mechanism *m3* by UPR1444, obtained from the PM6/AMBER free energy surfaces.

Distances	E:I	TS1	Int-3
**Active Site**			
N_{Lys745} - S_{SO2F}	2.40 ± 0.05	1.82 ± 0.02	1.76 ± 0.03
F_{SO2F} - S_{SO2F}	1.66 ± 0.05	1.75 ± 0.06	1.92 ± 0.12
N_{Lys745} - H_{Lys745}	1.04 ± 0.03	1.62 ± 0.08	2.58 ± 0.21
O_w - H_{Lys745}	2.05 ± 0.17	1.10 ± 0.04	0.99 ± 0.04
O_w - H_w	0.98 ± 0.04	1.20 ± 0.05	1.42 ± 0.14
O_{SO2F} - H_w	1.89 ± 0.15	1.36 ± 0.06	1.13 ± 0.08
**SO_2F and p-loop residues**			
O_{SO2F} - H_{Ala722}	3.91 ± 0.33	4.05 ± 0.31	3.94 ± 0.45
O_{SO2F} - H_{Phe723}	2.20 ± 0.21	2.21 ± 0.21	2.30 ± 0.23
O_{SO2F} - H_{Gly724}	2.24 ± 0.25	3.06 ± 0.25	2.23 ± 0.23

Table S6. Average distances (in Å) ± standard deviation for key states located along step 1 of the inhibition mechanism *m3* by UPR1433, obtained from the PM6/AMBER free energy surfaces.

Distances	E:I	TS1	Int-3
**Active Site**			
N_{Lys745} - S_{SO2F}	2.26 ± 0.05	1.79 ± 0.03	1.75 ± 0.03
F_{SO2F} - S_{SO2F}	1.68 ± 0.05	1.77 ± 0.06	1.83 ± 0.08
N_{Lys745} - H_{Lys745}	1.03 ± 0.03	2.16 ± 0.14	3.32 ± 0.18
O_w - H_{Lys745}	1.68 ± 0.23	1.03 ± 0.03	0.98 ± 0.04
O_w - H_w	0.99 ± 0.04	1.39 ± 0.09	1.68 ± 0.14
O_{SO2F} - H_w	1.90 ± 0.21	1.15 ± 0.06	1.02 ± 0.05
**SO_2F and p-loop residues**			
O_{SO2F} - H_{Ala722}	5.29 ± 0.34	5.46 ± 0.39	3.82 ± 0.63
O_{SO2F} - H_{Phe723}	4.00 ± 0.24	4.12 ± 0.22	2.66 ± 0.51
O_{SO2F} - H_{Gly724}	3.19 ± 0.30	3.32 ± 0.30	2.28 ± 0.33

Characterization of the TSs at the DFT/AMBER level of theory.

A micro-macro iteration optimization algorithm[26,27] was used to localize, optimize, and characterize the TS structures using a Hessian matrix containing all the coordinates of the QM subsystem at DFT/AMBER level of theory. The gradient norm of the remaining movable atoms was maintained lower than 0.01 kcal·mol^{-1}·Å^{-1}. Intrinsic reaction coordinates (IRCs) were traced down from located TSs to the valleys of the reactants, intermediates, and products in mass-weighted Cartesian coordinates. The hybrid M06-2X functional[15] with the standard 6-31+G(d,p) basis set[16] was used to treat the QM sub-set of atoms, as implemented in *Gaussian09* program.[28] The protein and solvent water molecules were treated with the AMBER,[6] as implemented in fDynamo,[29,30] and TIP3P force fields.[10]

Table S7. Cartesian coordinates (in Å) of QM atoms for TS1, Int-3, TS2, and E-I of the inhibition mechanism *m3* by XO44, optimized at M06-2X/6-31+G(d,p)/AMBER level.

TS1 (νi = 756.1i cm^{-1})				Int-3			
C	44.490757889882	36.412797508938	38.382063284073	C	44.943371538018	35.877259245297	38.196761173819
H	43.969896648577	36.481320111608	39.343425802689	H	44.450344630920	35.938938272349	39.171589313414
H	45.419402225017	36.989689195775	38.481957001223	H	45.879247798209	36.444501476266	38.280357348056
N	44.720882481008	34.998768419453	38.067416306318	N	45.172638183146	34.455013780954	37.909201445628
H	43.123726390648	34.473564240544	38.277506316099	H	42.999633321992	33.814406748777	37.913579412125
H	45.129502152682	34.870865020631	37.144787907768	H	45.669914918094	34.290539462257	37.038683764542
O	44.517149859127	33.955519843466	40.242881706662	O	44.691727393207	33.585894389335	40.094890168159
S	45.623186811397	33.807611430535	39.199242207838	S	45.934519077769	33.391184857434	39.165777147834
O	45.705335310009	32.854799839379	38.083780973762	O	46.193584256062	32.321447007076	38.192811320861
F	46.421677234125	32.722621905543	40.250153433782	F	46.562528952999	32.434267584553	40.433249215073
C	47.067594647048	34.868864578268	39.524461031777	C	47.304769101896	34.501871427455	39.576327968734
C	47.870743172945	35.306418686972	38.477790111349	C	48.121654382074	34.942738398068	38.542181388304
C	49.047207256549	36.008560534753	38.764565451355	C	49.239438268049	35.716138917389	38.848025895108
H	49.676901642782	36.324613916807	37.936876604703	H	49.890183682424	36.032023156336	38.038253129578
H	47.616840339749	35.060977595224	37.450069382249	H	47.917329937468	34.653221574070	37.515454576372
C	47.439495396470	35.093828680269	40.847335897558	C	47.591082283565	34.803398938924	40.903186705039
H	46.856666380104	34.667174286319	41.657876194591	H	46.994108580489	34.380660476511	41.704378292615
C	48.601847924924	35.813690196358	41.120716697978	C	48.692117570848	35.609282366832	41.192065244133
H	48.881210771636	35.968048169975	42.158301782621	H	48.914933639782	35.832447982833	42.230734394076
C	49.427692615939	36.273353982168	40.087211763464	C	49.532323657119	36.063966743361	40.172207903881
C	50.752905918850	36.968389309240	40.422415922500	C	50.817528449469	36.814351884636	40.502663584820
H	50.981341902824	36.829783648898	41.484771636890	H	51.069239334621	36.687914880625	41.560146128270
O	42.485730375946	34.099903086108	38.984403521409	O	42.624510095336	33.559522940860	38.770555215365
H	43.326056757652	33.994562437229	39.728521977021	H	43.777855811976	33.595898992076	39.543929007072

TS2 (νi = 623.4i cm^{-1})	E-I
C 44.142491676394 36.366166117954 38.436208578268	C 44.152886174273 36.386793948953 38.404883504789
H 43.481415619714 36.151218552531 39.280997891202	H 43.523376926914 36.220406584822 39.283176875591
H 44.915678059921 37.080177599819 38.739019757023	H 44.965521437069 37.078250109634 38.649072759954
N 44.824177159724 35.146262628994 37.949661179419	N 44.766970286239 35.134107944082 37.916746564139
H 41.960212631889 33.708223301221 39.593715935944	H 42.047857071957 33.823778912135 39.691871524628
H 44.199859275520 34.572016671103 37.384206818287	H 44.084424411516 34.530972216329 37.452681201751
O 44.610239018811 33.936786423371 40.110372196128	O 44.920539278197 33.873470290398 40.187204198718
S 45.640257703239 34.031716893820 38.913079582507	S 45.624129286245 34.127039597053 38.924286867093
O 45.801201953258 32.969882325906 37.937312044363	O 45.990146000414 32.995267440877 38.088651591611
F 46.478225140214 32.725118568439 40.258930370974	F 45.883915803193 31.281477441692 40.511855403609
C 47.115543049606 34.957226033088 39.400211922111	C 47.114511905391 35.029069679676 39.352912347865
C 47.972021050882 35.362676798182 38.382687191390	C 48.002424652934 35.411753685738 38.347397937317
C 49.144712151204 36.043396762237 38.719095621143	C 49.168531464799 36.084707846892 38.698976354652
H 49.807632764368 36.356075885937 37.918756263652	H 49.842932576857 36.408193156474 37.912693767371
H 47.742293236313 35.126404931086 37.347907871752	H 47.783620657802 35.174603792652 37.310031261186
C 47.435453462788 35.166812171660 40.739226427142	C 47.419917495839 35.230132310020 40.695134094809
H 46.819870532235 34.760257274869 41.533232422372	H 46.757192467609 34.861976973564 41.471617463836
C 48.606461648752 35.855840857636 41.057603143320	C 48.605090717233 35.887365364803 41.036889573419
H 48.850450297687 36.001998623016 42.105946291342	H 48.840895893614 36.035856077504 42.086684119484
C 49.474820156083 36.305188046759 40.054977660991	C 49.481329127777 36.330321172712 40.045165703351
C 50.783473738518 37.014149604312 40.422416365254	C 50.785673200043 37.025378713177 40.424983279216
H 50.982410928139 36.903751138853 41.494006821978	H 50.982148990550 36.919241868095 41.496635451748
O 41.781812608728 34.444545380329 38.999855601091	O 41.848360197535 34.524743809263 39.062215336522
H 45.105388717100 33.236640626146 40.663715168238	H 45.412477027305 32.060995003116 40.710384351298

Table S8. Key distances (in Å) for the states located along of the inhibition mechanism *m3* by XO44, optimized at M06-2X/6-31+G(d,p)/AMBER level of theory.

Distances	E:I	TS1	Int-3	TS2	E-I
**Active Site**					
N_{Lys745} - S_{SO2F}	2.76	1.87	1.72	1.68	1.66
F_{SO2F} - S_{SO2F}	1.59	1.71	1.83	2.05	3.27
N_{Lys745} - H_{Lys745}	1.02	1.69	3.64	3.60	3.50
O_w - H_{Lys745}	2.06	1.02	0.96	0.96	0.96
O_w - H_w	0.96	1.13	3.68	3.91	4.64
O_{SO2F} - H_w	2.12	1.30	0.98	1.02	1.95
F_{SO2F} - H_w	4.28	3.39	1.83	1.52	0.93
**SO_2F and p-loop residues**					
O_{SO2F} - H_{Ala722}	3.23	4.41	4.62	4.59	4.44
O_{SO2F} - H_{Phe723}	1.99	2.83	2.99	2.99	2.96
O_{SO2F} - H_{Gly724}	2.07	1.98	2.10	2.11	2.17

Table S9. Cartesian coordinates (in Å) of QM atoms for TS1, Int-3, and E-I of the inhibition mechanism *m3* by UPR1444, optimized at M06-2X/6-31+G(d,p)/AMBER level.

TS1 (vi = 1288.8i cm^{-1})	Int-3
C 48.587650844050 50.400306297637 36.598398448009	C 48.590164262813 50.394964155242 36.609461052808
H 47.907512706242 49.549408426810 36.695018196598	H 47.908789457926 49.542003398975 36.662404210150
H 48.998334071082 50.566197254486 37.599171360452	H 48.952959168089 50.543870027900 37.633588656765
N 49.619606297694 50.061657673472 35.613533147670	N 49.663918188580 50.074519730254 35.664694551131
H 50.360478386493 50.759035947032 35.598942679108	H 50.393195951008 50.781849415545 35.662516689712
H 48.890497807067 49.923129280435 34.202675048589	H 48.726462860542 50.059329007060 33.834000610416
N 50.691259121082 49.580504585362 42.446777412593	N 50.679549817792 49.582239638387 42.465056232573
H 50.977577516911 49.574072263430 43.419695155979	H 50.958205193769 49.584042484467 43.440259333861
C 51.103625447528 48.359945664032 41.774105562913	C 51.120013618622 48.368618880856 41.795646457499
H 50.634770521150 47.440814741715 42.156595045755	H 50.676709037974 47.439539573110 42.183597628709
C 50.931356634183 48.379149738556 40.260985066837	C 50.943188246488 48.384531644436 40.287094774955
C 51.286203846727 49.504127911151 39.508239389741	C 51.281677487294 49.522260972806 39.548018186581
C 51.172311928768 49.504304710858 38.122135092605	C 51.167584316550 49.531940604147 38.164639826510
H 51.508096701817 50.356650973999 37.538367835110	H 51.483085943805 50.396775531859 37.588788255820
H 51.650287962733 50.385080220168 40.024531543843	H 51.628602070023 50.404150661888 40.073962835138
C 50.423151976819 47.262343252109 39.588616046349	C 50.463303861705 47.260162487208 39.608414753329
H 50.145123584953 46.371347344047 40.144683152315	H 50.203756263684 46.360370778846 40.159100503545
C 50.267342242523 47.254046430466 38.201141254509	C 50.314048910752 47.258298835068 38.220483741311
H 49.886045906466 46.376834025274 37.690704712270	H 49.956396857292 46.376445407571 37.700666185026
C 50.641693417751 48.384187895572 37.487361659216	C 50.661987561548 48.405707809274 37.520664836519
S 50.554928179886 48.401072234796 35.691982854313	S 50.557707007062 48.482502531906 35.731855746282
O 51.663955013736 49.011981208868 34.958722240664	O 51.678012705340 49.038949561099 34.975679103753
F 51.331442959000 46.873357263184 35.744500088331	F 51.285469217946 46.912646639103 35.745733628641
O 49.350914845843 47.690401528049 35.045931701822	O 49.316677141535 47.741502047975 35.108349496315
O 48.599815010325 49.274252769898 33.441682002463	O 48.553877720706 49.260571921106 33.307675026931
H 47.661399572296 49.294119490185 33.141298160192	H 47.622375074969 49.235957825909 32.982324813755
H 48.892935848523 48.357557034836 34.143343777758	H 48.919110656927 48.331729427090 34.339445116463

E-I
C 49.094094814846 50.440959155870 36.414831810537
H 48.458445915794 49.551323486832 36.390621120070
H 49.303576418923 50.648550064900 37.460677457721
N 50.374978449926 50.209270900653 35.721388442564
H 50.341394687326 50.419939999028 34.724413268204
H 49.363220152920 44.304224479933 34.775438965972
N 50.455379197174 48.781812558821 42.857162027033
H 50.972597573706 49.091907484534 43.665142051941
C 51.256926841117 48.155729084669 41.808730472854
H 51.426676203811 47.100392353624 42.044450785188
C 50.864770210248 48.262982678965 40.352453886841
C 51.294052238327 49.437719038756 39.717160776586
C 51.346476103438 49.525568274796 38.346107820648
H 51.763481556394 50.387794155046 37.835507470787
H 51.652794339890 50.257811738986 40.328858763206
C 50.391383624221 47.197480702843 39.593989550163
H 50.086770826361 46.272536742282 40.074888122103
C 50.345414611363 47.300868185354 38.200493840375
H 49.963904555308 46.494785611860 37.585536040647
C 50.843560134351 48.450657368150 37.612899344656
S 50.884388664243 48.601065471876 35.867254335729
O 52.254440225751 48.526018751649 35.401588248740
F 51.997081812758 45.804640239250 34.968868511701
O 49.880684848673 47.726699134937 35.268922823129
O 49.587950046283 45.121005203007 35.254753614530
H 49.206645420489 45.878732067029 34.790579483063
H 51.118200468417 45.466195152271 35.169440562579

Table S10. Key distances (in Å) for the states located along of the inhibition mechanism *m3* by UPR1444, optimized at M06-2X/6-31+G(d,p)/AMBER level of theory.

Distances	E:I	TS1	Int-3	E-I
**Active Site**				
N_{Lys745} - S_{SO2F}	2.85	1.91	1.83	1.64
F_{SO2F} - S_{SO2F}	1.61	1.71	1.73	3.14
N_{Lys745} - H_{Lys745}	1.02	1.59	2.06	6.07
O_w - H_{Lys745}	2.83	1.04	0.97	0.97
O_w - H_w	0.96	1.19	1.44	1.57
O_{SO2F} - H_w	4.90	1.21	1.05	2.58
F_{SO2F} - H_w	6.87	3.27	3.10	0.96
**SO_2F and p-loop residues**				
O_{SO2F} - H_{Ala722}	3.84	4.50	4.53	3.85
O_{SO2F} - H_{Phe723}	2.14	2.50	2.52	2.19
O_{SO2F} - H_{Gly724}	2.15	2.05	2.03	1.86

Table S11. Cartesian coordinates (in Å) of QM atoms for TS1, TS2, Int-3 and E-I of the inhibition mechanism *m3* by UPR1433, optimized at M06-2X/6-31+G(d,p)/AMBER level.

TS1 (νi = 880.7i cm^{-1})	Int-3
C 48.560141934599 50.220869347812 35.882217719521	C 48.559986122637 50.223360387024 35.915240821422
H 47.659013249379 49.993259247099 35.305812569535	H 47.674200709551 49.998673896709 35.312842276792
H 48.539244932642 49.572826649878 36.761111653783	H 48.500293928583 49.590207255482 36.802807133946
N 49.730537148442 49.870596569871 35.054925191906	N 49.732483057569 49.827283760679 35.137058085605
H 49.548827613770 49.104738157513 33.709126143512	H 49.224075632141 48.281812999905 33.249150383288
H 50.112884594557 50.747346899174 34.692421641145	H 50.021342426720 50.601196504488 34.537834789240
O 51.784874142870 48.737433638386 34.501861417138	O 51.737972322084 48.819745453127 34.485322626455
S 51.299160982503 49.222378535345 35.879345182543	S 51.232896272331 49.304139791895 35.915665766305
O 51.578132844321 50.503401004939 36.543676718115	O 51.574502029935 50.551496800043 36.611649987035
F 52.763514318262 48.513875052919 36.494054638111	F 52.746098203789 48.563522944103 36.418481765643
C 50.504175637738 47.947742086537 36.863126518742	C 50.509562897836 47.982333981640 36.889561545067
C 50.200375162350 48.271094494622 38.177941588512	C 50.197755507120 48.289004236908 38.204155080657
H 50.358982895075 49.290025825418 38.517579235978	H 50.347898266696 49.304679939241 38.556818390140
C 50.332643341679 46.666194674920 36.357935416930	C 50.340192953199 46.706648014179 36.368242641047
H 50.580199268390 46.446078334968 35.325158842481	H 50.596869805577 46.497418302332 35.335398517468
C 49.814285262818 45.685776236863 37.204343811756	C 49.808756876404 45.718299891857 37.196128932471
H 49.647086006262 44.681645501927 36.832865688297	H 49.636572687775 44.720883611147 36.809083589350
C 49.471051139293 45.993058305800 38.518671715969	C 49.452183323640 46.010847790766 38.510969615685
H 49.034885184029 45.230256668762 39.153633039040	H 48.998411631745 45.245595213044 39.130445968640
C 49.662741922891 47.285642908872 39.013110905779	C 49.651057773285 47.294340340112 39.022152105210
C 49.159778047833 47.647898495702 40.382985294153	C 49.145856836872 47.651296155683 40.389914525713
O 48.222039383170 47.035093907565 40.898521761695	O 48.202161094494 47.044054933460 40.901126435078
N 49.748221469738 48.722257102341 40.960006221433	N 49.743351204137 48.719776188263 40.966488739571
H 50.639417803174 49.039569076492 40.612323269857	H 50.638193274343 49.028957671430 40.621533497496
O 50.067352738726 48.711675423396 32.862918880370	O 49.970257155229 48.511876212338 32.660374126812
H 51.010888846946 48.716512467277 33.544207467571	H 51.013398185113 48.831895852381 33.770876314630
H 50.103206523761 49.367529719365 32.153712245177	H 49.681195539748 49.203490287701 32.055153332527

TS2 (νi = 508.5i cm^{-1})				E-I			
C	49.970776016571	51.691027147243	36.797134561853	C	49.924360251161	51.731170138047	36.827970973940
H	49.089108146330	51.954944701117	37.381742950418	H	49.065002226415	52.028151713855	37.425434685603
H	50.813122816361	51.587331555858	37.482754921145	H	50.779398672619	51.575817495245	37.488641122420
N	49.714076669262	50.372406022210	36.204056035699	N	49.600865265149	50.448798396610	36.196951410751
H	49.415451150357	46.116673185873	33.784545411611	H	49.483841755212	46.045738955331	33.713397517565
H	49.087503735753	50.331460292447	35.409059039303	H	49.043637660113	50.484390150251	35.349962761687
O	50.577771765896	48.839910116340	34.614729088680	O	50.607636590802	48.730490773519	34.704948087472
S	50.931973777005	49.228467538294	36.093690901289	S	50.756199437770	49.259086827085	36.055046537173
O	52.108449258422	49.930918113616	36.553836273539	O	52.018180088462	49.807970157689	36.526105257395
F	52.310928199806	47.642058506494	35.501510442943	F	52.323167278593	46.387437004860	35.003704271559
C	50.382050756157	47.950544806437	37.220587566426	C	50.265824762480	48.020443176205	37.235672344081
C	50.019825319941	48.406775343644	38.481365518495	C	49.976698987284	48.452632236370	38.522724364236
H	50.017669712861	49.469677491239	38.699985158271	H	49.958632722616	49.514098163061	38.749071411385
C	50.323281798802	46.605667423778	36.869297249682	C	50.198397798110	46.677166037618	36.893212225530
H	50.628301550411	46.283669480881	35.881659637973	H	50.371079744205	46.374125917101	35.875258027166
C	49.884320527557	45.696897587015	37.830719222707	C	49.884565471007	45.736943165120	37.874657960616
H	49.809525476760	44.645312660216	37.579633707453	H	49.834075905183	44.684983273044	37.620667861689
C	49.525286172172	46.128752801683	39.102993676187	C	49.588769118784	46.153045458406	39.164205584187
H	49.179575216484	45.408069716899	39.833531979988	H	49.314764817133	45.428269296785	39.920980205773
C	49.595859078347	47.484466023959	39.437719064866	C	49.617577957420	47.513418882122	39.483619462546
C	49.066578146382	47.965046606385	40.756474459736	C	49.093801490717	47.986372692196	40.796904956662
O	48.175030435792	47.341834864096	41.331980465995	O	48.209643657651	47.362064144310	41.382530073974
N	49.569110343200	49.142876095007	41.209967129732	N	49.585517091384	49.173348912176	41.221585975609
H	50.475833959170	49.435528195808	40.880446967287	H	50.480623593763	49.484296295222	40.879492586129
O	50.373252943076	46.207357165822	33.621330809989	O	50.378788625342	46.324201406089	33.438232063701
H	51.157566295284	48.024999323351	34.443429845187	H	51.584834148552	46.351356310077	34.371901841015
H	50.497933770689	46.024279474850	32.683794278179	H	50.302026733419	47.277057055430	33.286634325806

Table S12. Key distances (in Å) for the states located along of the inhibition mechanism *m3* by UPR1433, optimized at M06-2X/6-31+G(d,p)/AMBER level of theory.

Distances	E:I	TS1	Int-3	TS2	E-I
**Active Site**					
N_{Lys745} - S_{SO2F}	2.49	1.89	1.77	1.67	1.66
F_{SO2F} - S_{SO2F}	1.64	1.74	1.76	2.18	3.44
N_{Lys745} - H_{Lys745}	1.03	1.56	2.49	4.90	5.06
O_w - H_{Lys745}	2.46	1.07	0.98	0.98	0.98
O_w - H_w	0.97	1.16	1.56	2.14	1.53
O_{SO2F} - H_w	1.97	1.23	1.02	1.01	2.59
F_{SO2F} - H_w	4.31	3.44	3.18	1.61	0.97
**SO_2F and p-loop residues**					
O_{SO2F} - H_{Ala722}	5.64	5.67	5.71	5.14	5.08
F_{SO2F} - H_{Ala722}	3.37	3.38	3.44	3.25	3.99
O_{SO2F} - H_{Phe723}	4.14	4.17	3.54	3.55	3.54
F_{SO2F} - H_{Phe723}	2.45	2.46	2.42	1.79	2.02
O_{SO2F} - H_{Gly724}	3.51	3.50	3.53	2.79	2.87
F_{SO2F} - H_{Gly724}	2.42	2.55	2.50	3.03	2.73

References.

[1] Zhao, Q.; Ouyang, X.; Wan, X.; Gajiwala, K. S.; Kath, J. C.; Jones, L. H.; Burlingame, A. L.; Taunton, J. Broad-spectrum kinase profiling in live cells with lysine-targeted sulfonyl fluoride probes. *J. Am. Chem. Soc.*, **2017**, *139*, 680-685.

[2] MacroModel, Schrödinger, LLC, New York, NY, 2019.

[3] Harder, E.; Damm, W.; Maple, J.; Wu, C.; Reboul, M.; Xiang, J. Y.; Wang, L.; Lupyan, D.; Dahlgren, M. K.; Knight, J. L.; Kaus, J. W.; Cerutti, D. S.; Krilov, G.; Jorgensen, W. L.; Abel, R.; Friesner, R. A. OPLS3: a force field providing broad coverage of drug-like small molecules and proteins. *J. Chem. Theory Comput.*, **2016**, *12*, 281-296.

[4] Ferlenghi, F.; Scalvini, L.; Vacondio, F.; Castelli, R.; Bozza, N.; Marseglia, G.; Rivara, S.; Lodola, A.; La Monica, S.; Minari, R.; Petronini, P. G.; Alfieri, R.; Tiseo, M.; Mor, M. A sulfonyl fluoride derivative inhibits EGFRL858R/T790M/C797S by covalent modification of the catalytic lysine. *Eur. J. Med. Chem.*, **2021**, *225*, 113786.

[5] MacroModel, Schrödinger, LLC, New York, NY, 2019.

[6] Case, D. A.; Aktulga, H. M.; Belfon, K.; Ben-Shalom, I. Y.; Berryman, J. T.; Brozell, S. R.; Cerutti, D. S., Cheatham, I. T. E.; Cisneros, G. A.; Cruzeiro, V. W. D.; Darden, T. A.; Duke, R. E.; Giambasu, G.; Gilson, M. K.; Gohlke, H.; Goetz, A. W.; Harris, R.; Izardi, S.; Izmailov, S. A.; Kasavajhala, K.; Kaymak, M. C.; King, E.; Kovalenko, A.; Kurtzman, T.; Lee, T. S.; LeGrand, S.; Li, P.; Lin, C.; Liu, J.; Luchko, T.; Luo, R.; Machado, M.; Man, V.; Manathunga, M.; Merz, K. M.; Miao, Y.; Mikhailovskii, O.; Monard, G.; Nguyen, H.; O'Hearn, K. A.; Onufriev, A.; Pan, F.; Pantano, S.; Qi, R.; Rahnamoun, A.; Roe, D. R.; Roitberg, A.; Sagui, C.; Schott-Verdugo, S.; Shajan, A.; Shen, J.; Simmerling, C. L.; Skrynnikov, N. R.; Smith, J.; Swails, J.; Walker, R. C.; Wang,

J.; Wei, H.; Wolf, R.M.; Wu, X.; Xiong, Y.; Xue, Y.; York, D. M.; Zhao, S.; Kollman, P. A. AMBER 2020, 2020.

[7] Duan, Y.; Wu, C.; Chowdhury, S.; Lee, M. C.; Xiong, G.; Zhang, W.; Yang, R.; Cieplak, P.; Luo, R.; Lee, T.; Caldwell, J.; Wang, J.; Kollman, P.; A point-charge force field for molecular mechanics simulations of proteins based on condensed-phase quantum mechanical calculations. *J. Comput. Chem.*, **2003**, *24*, 1999-2012.

[8] Lee, M. C.; Duan, Y. Distinguish protein decoys by using a scoring function based on a new AMBER force field, short molecular dynamics simulations, and the generalized born solvent model. *Proteins*, **2004**, *55*, 620-634.

[9] Wang, J.; Wolf, R. M.; Caldwell, J. W.; Kollman, P. A.; Case, D. A. Development and testing of a general amber force field. *J. Comput. Chem.*, **2004**, *25*, 1157-1174.

[10] Jorgensen, W. L.; Chandrasekhar, J.; Madura, J. D.; Impey, R. W.; Klein. M. L. Comparison of simple potential functions for simulating liquid water. *J. Chem. Phys.*, **1983**, *79*, 926-935.

[11] Olsson, M. H.; Søndergaard, C. R.; Rostkowski, M.; Jensen, J. H. PROPKA3: consistent treatment of internal and surface residues in empirical pK_a predictions. *J. Chem. Theory Comput.*, **2011**, *7*, 525-537.

[12] Stewart. J. J.; Optimization of parameters for semiempirical methods V: modification of NDDO approximations and application to 70 elements. *J. Mol. Model.*, **2007**, *13*, 1173-1213.

[13] Dewar, M. J. S.; Zoebisch, E. G.; Healy, E. F.; Stewart, J. J. P. Development and use of quantum mechanical molecular models. 76. AM1: a new general purpose quantum mechanical molecular model. *J. Am. Chem. Soc.*, **1985**, *107*, 3902-3909.

[14] Stewart, J. J.; Optimization of parameters for semiempirical methods I. method. *Method J. Comput. Chem.*, **1989**, *10*, 209-220.

[15] Zhao, Y.; Truhlar, D. G. The M06 suite of density functionals for main group thermochemistry, thermochemical kinetics, noncovalent interactions, excited states, and transition elements: two new functionals and systematic testing of four M06-class functionals and 12 other functionals. *Theor. Chem. Acc.*, **2008**, *120*, 215-241.

[16] Wiberg, K. B. Ab Initio molecular orbital theory by W. J. Hehre, L. Radom, P. v. R. Schleyer, J. A. Pople, John Wiley, New York, 1986, pp 548.

[17] Moller, C.; Plesset, M. S. Note on an approximation treatment for many-electron systems. *Phys. Rev.*, **1934**, *46*, 618.

[18] Pople, J. A.; Seeger, R.; Krishnan, R. Variational configuration interaction methods and comparison with perturbation theory. *Int. J. Quantum Chem. Symp.*, **1977**, *11*, 149.

[19] Bartlett, R. J. Many-body perturbation theory for quasiparticle energies. *Annu. Rev. Phys. Chem.*, **1981**, *32*, 359.

[20] Frisch, M. J.; Trucks, G. W.; Schlegel, H. B.; Scuseria, G. E.; Robb, M. A.; Cheeseman, J. R.; Scalmani, G.; Barone, V.; Petersson, G. A.; Nakatsuji, H.; Li, X.; Caricato, M.; Marenich, A. V.; Bloino, J.; Janesko, B. G.; Gomperts, R.; Mennucci, B.; Hratchian, H. P.; Ortiz, J. V.; Izmaylov, A. F.; Sonnenberg, J. L.; Williams-Young, D.; Ding, F.; Lipparini, F.; Egidi, F.; Goings, J.; Peng, B.; Petrone, A.; Henderson, T.; Ranasinghe, D.; Zakrzewski, V. G.; Gao, J.; Rega, N.; Zheng, G.; Liang, W.; Hada, M.; Ehara, M.; Toyota, K.; Fukuda, R.; Hasegawa, J.; Ishida, M.; Nakajima, T.; Honda, Y.; Kitao, O.; Nakai, H.; Vreven, T.; Throssell, K.; Montgomery, J. A.; Peralta, Jr., J. E.; Ogliaro, F.; Bearpark, M. J.; Heyd, J. J.; Brothers, E. N.; Kudin, K. N.; Staroverov, V.

N.; Keith, T. A.; Kobayashi, R.; Normand, J.; Raghavachari, K.; Rendell, A. P.; Burant, J. C.; Iyengar, S. S.; Tomasi, J.; Cossi, M.; Millam J. M.; Klene, M.; Adamo, C.; Cammi, R., Ochterski, J. W., Martin, R. L., Morokuma, K.; Farkas, O.; Foresman, J. B.; Fox, D. J. Gaussian 16 (Revision B.01), 2016.

[21] Marenich, A. V.; Cramer, C. J.; Truhlar, D. G. Universal solvation model based on solute electron density and on a continuum model of the solvent defined by the bulk dielectric constant and atomic surface tensions. *J. Phys. Chem. B.*, **2009**, *113*, 6378.

[22] Marcos-Alcalde, I.; Setoain, J.; Mendieta-Moreno, J. I.; Mendieta, J.; Gómez-Puertas, P. MEPSA: minimum energy pathway analysis for energy landscapes. *Bioinformatics*, **2015**, *31*, 3853-3855.

[23] Torrie, G. M.; Valleau, J. P. Nonphysical sampling distributions in monte carlo free-energy estimation: umbrella sampling. *J. Comput. Phys.*, **1977**, *23*, 187-199.

[24] Kumar, S.; Bouzida, D.; Swendsen, R. H.; Kollman, P. A.; Rosenberg, J. M. The Weighted Histogram Analysis method for free-energy calculations on biomolecules. I. the method. *J. Comput. Chem.*, **1992**, *13*, 1011-1021.

[25] Grossfield, A. “WHAM: the weighted histogram analysis method”, version 2.0.10, http://membrane.urmc.rochester.edu/wordpress/?page_id=126).

[26] Turner, A. J.; Moliner, V.; Williams, I. H. Transition-state structural refinement with GRACE and CHARMM: flexible QM/MM modeling for lactate dehydrogenase. *Phys. Chem. Chem. Phys.*, **1999**, *1*, 1323-1331.

[27] Martí, S.; Moliner, V.; Tuñón, I. Improving the QM/MM description of chemical processes: a dual level strategy to explore the potential energy surface in very large systems. *J. Chem. Theory Comput.*, **2005**, *1*, 1008-1016.

[28] Frisch, M. J.; Trucks, G. W.; Schlegel, H. B.; Scuseria, G. E.; Robb, M. A.; Cheeseman, J. R.; Scalmani, G.; Barone, V.; Petersson, G. A.; Nakatsuji, H.; Li, X.; Caricato, M.; Marenich, A. V.; Bloino, J.; Janesko, B. G.; Gomperts, R.; Mennucci, B.; Hratchian, H. P.; Ortiz, J. V.; Izmaylov, A. F.; Sonnenberg, J. L.; Williams-Young, D.; Ding, F.; Lipparini, F.; Egidi, F.; Goings, J.; Peng, B.; Petrone, A.; Henderson, T.; Ranasinghe, D.; Zakrzewski, V. G.; Gao, J.; Rega, N.; Zheng, G.; Liang, W.; Hada, M.; Ehara, M.; Toyota, K.; Fukuda, R.; Hasegawa, J.; Ishida, M.; Nakajima, T.; Honda, Y.; Kitao, O.; Nakai, H.; Vreven, T.; Throssell, K.; Montgomery, J. A.; Peralta, Jr., J. E.; Ogliaro, F.; Bearpark, M. J.; Heyd, J. J.; Brothers, E. N.; Kudin, K. N.; Staroverov, V. N.; Keith, T. A.; Kobayashi, R.; Normand, J.; Raghavachari, K.; Rendell, A. P.; Burant, J. C.; Iyengar, S. S.; Tomasi, J.; Cossi, M.; Millam J. M.; Klene, M.; Adamo, C.; Cammi, R., Ochterski, J. W., Martin, R. L., Morokuma, K.; Farkas, O.; Foresman, J. B.; Fox, D. J. Gaussian 09 (Revision A.01), 2009.

[29] Field, M. J.; Albe, M.; Bret, C.; Proust-De Martin, F.; Thomas, A. The dynamo library for molecular simulations using hybrid quantum mechanical and molecular mechanical potentials. *J. Comp. Chem.*, **2000**, *21*, 1088-1100.

[30] Krzeminska, A.; Paneth, P.; Moliner, M.; Swiderek, K. Binding isotope effects as a tool for distinguishing hydrophobic and hydrophilic binding sites of HIV-1 RT. *J. Phys. Chem. B*, **2015**, *119*, 917-927.
